# Growing bacterial colonies harness emergent genealogical demixing to regulate organizational entropy

**DOI:** 10.1016/j.bpr.2024.100175

**Published:** 2024-08-26

**Authors:** Garima Rani, Anupam Sengupta

**Affiliations:** 1 Physics of Living Matter Group, Department of Physics and Materials Science, University of Luxembourg, 162a Avenue de la Faïencerie, Luxembourg City, Grand Duchy of Luxembourg; 2 Institute for Advanced Studies, University of Luxembourg, 2 Avenue de l’Université, Esch-sur-Alzette, Grand Duchy of Luxembourg

## Abstract

Spatiotemporal organization of individuals within growing bacterial colonies is a key determinant of intraspecific interactions and colony-scale heterogeneities. The evolving cellular distribution, in relation to the genealogical lineage, is thus central to our understanding of bacterial fate across scales. Yet, how bacteria self-organize genealogically as a colony expands has remained unknown. Here, by developing a custom-built label-free algorithm, we track and study the genesis and evolution of emergent self-similar genealogical enclaves, whose dynamics are governed by biological activity. Topological defects at enclave boundaries tune finger-like morphologies of the active interfaces. The Shannon entropy of cell arrangements reduce over time; with faster-dividing cells possessing higher spatial affinity to genealogical relatives, at the cost of a well-mixed, entropically favorable state. Our coarse-grained lattice model demonstrates that genealogical enclaves emerge due to an interplay of division-mediated dispersal, stochasticity of division events, and cell-cell interactions. The study reports so-far hidden emergent self-organizing features arising due to entropic suppression, ultimately modulating intraspecific genealogical distances within bacterial colonies.

## Graphical abstract

**Figure:**
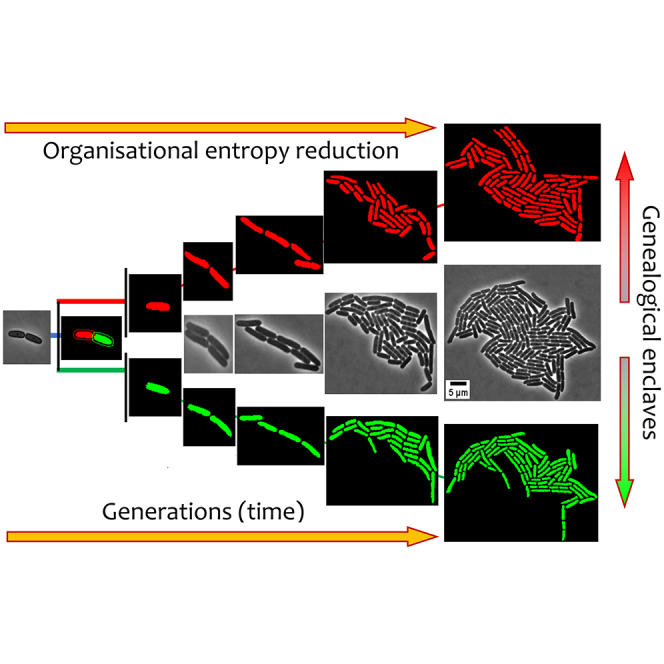

## Why it matters

Spatiotemporal distribution of bacteria has far-reaching ramifications in the ecology and evolution of bacterial species and their consortia. Many species are surface associated, yet how they distribute genealogically, i.e., how daughter cells distribute in relation to their mother cells, specifically during the early stages of biofilm formation, remains unknown. By analyzing expanding colonies using a custom-built label-free algorithm, we track bacterial growth, revealing distinct self-similar genealogical enclaves that intermix over time. While biological activity determines their intermixing dynamics, emergent topological defects at the interfaces mediate the finger-like morphology of interfacial domains. Our results demonstrate that proximity to kith and kin—both spatial and genealogical—is intrinsically encoded in growth from an early developmental stage, signifying its role in mediating fitness and viability.

## Introduction

Bacterial colonies, comprising one or several species, execute a range of ecological and biomedical functions (1,2) via cell-cell communication strategies (3,4), collective behavior (5), response to stresses (6), and self-regulation of biophysical traits (7,8). A key factor underpinning the evolutionary success of bacteria is their ability to form structured communities such as biofilms, wherein spatial organization of cells determines individual and population scale behavior (9,10). The temporal evolution of such structured living systems is inherently coupled to the ability of individuals to disperse across different physical and timescales, allowing for reconfiguration of cell-cell interaction networks and adjustment of local composition of biofilms. Consequently, the evolution of the spatial structure of microbial agglomerations is central to the cell and lineage fate. Most naturally occurring bacterial communities comprise multiple species, which points to their role in promoting colonization and survivability. Spatial distribution of cell types and lineages thereof have been frequently found to remain compositionally demixed, characterized by clustering of cells of one type, which likely confers physiological and metabolic benefits in terms of cooperative sharing of available resources, production and exchange of metabolites, and selective amplification of beneficial traits, including more effective absorption of nutrients (9,11). Two fundamental questions arise in this context: how do cells and their lineages spatially distribute as they grow within a monospecific bacterial colony; and how does the spatial architecture evolve over time, particularly in the early stages of colony formation (7,8).

Cells in bacterial colonies show several remarkable features of self-organization (12,13,14) and the importance of structural order and topological attributes of cell organization in optimizing growth of such colonies has been highlighted (7,15). In particular, singularities in local ordering of cells in colonies, referred to as topological defects, have been implicated in driving the formation of layers in biofilms for both motile and sessile bacteria (16,17), which marks a significant step in biofilm growth and maturation. Topological defects have also been shown to regulate several other fundamental features of biofilm growth and propagation such as navigation (18), sporulation (19), and nutrient uptake (20). Such defects are common in the periphery of bacterial colonies driving advancing fronts but they are also regularly observed in the interior of the colonies, seemingly at random (21). Furthermore, as with any structured living community, a key determinant of its resilience are interaction between its constituents (22,23). Arguably, the most meaningful interactions in microbial communities are between close relatives on one hand and spatial neighbors on the other hand (24,25). Therefore, a mechanistic understanding of the growth of bacterial communities and designing on-demand effective strategies to limit the growth of deleterious biofilms rely on our understanding of cellular organization spatially, and in relation to their lineage kin. For such studies, it is essential to be able to track cells as they grow and divide over generations to form progeny chains. Simultaneous tracking of the cells and their lineage over time is a key challenge in addressing these questions. In general, cell tracking thus far has been largely done by fluorescent-based tracking methods (26), resulting in several insights into the spatial organization of cells in colonies (14,27,28). However, these methods are inadequate for long-term investigations due to fluorescent bleaching, and potential chemo- and phototoxic impacts on the cellular physiology. Consequently, such studies will benefit from label-free tracking techniques (for instance, based on image analysis), ultimately allowing accurate detection of emerging spatial patterns and subtle differences therein, arising due to varying growth and local environmental conditions.

In this work, using a custom-built label-free algorithm, we track single cells transforming into sessile colonies, and study the genesis and evolution of *genealogical enclaves*, distinct subcolonies comprising descendants of individual bacterial cells, as the colony grows onward from a single cell. The analyses of the emerging intracolony dynamics explains how progeny cells form enclaves within the colony, displaying spatial affinity for genealogically close relatives, at the cost of an entropically favorable option of intermixing. The dynamics of emerging enclaves, along with those of the topological defects in the colonies, display a high degree of self-similarity with the colonies at large on several key phenotypic traits over multiple division cycles, an intriguing feature common in fractals, including natural objects as disparate as coastlines and vegetables such as broccoli. These far-from-equilibrium arrangements of cells in such colonies represents a distinct yet unifying feature of pattern formation in nature, uncovering several so-far hidden features of emergent organization within bacterial colonies as presented below.

## Materials and methods

### Bacterial cultures

The primary strain used in this work is a Gram-negative bacteria *Escherichia coli* strain, namely NCM3722 delta-motA. The second species considered in the study is *Vibrio cholerae*, to understand the effect of cell shape (specifically low aspect ratio) on the spatial distribution of progeny chains. The cells were first streaked on a standard lysogeny broth (LB) agar plate and grown by setting the incubator at the appropriate temperature. After a day of growth in the plate, single isolated colonies were identified and picked using a inoculation loop and transferred to liquid LB medium in a shaker and shaken at 170 rpm. The cells were allowed to grow and divide in the shaker until late exponential phase (with regular subsampling done to track the cell growth over time by measuring the optical density), after which the cells were transferred to fresh LB medium at a dilution factor of 1:1000 (ratio of bacterial suspension to medium) and grown for ∼1.5−2 h (early exponential phase). Around ∼1−1.5μL droplets were then inoculated onto a thin layer of specially designed substrate. At least three biological replicates for each case were considered in this study and, further, different locations on the agarose substrate were imaged, thus utilizing multiple technical replicates from the same sample. More details of the experimental protocol may be found in (7).

### Agar pad preparation and time-lapse imaging

Low melting agarose was mixed with LB medium and the gel-like solution was poured into the Gene-frame (Thermo Scientific, Germany, Gene Frame with thickness ≈0.25 mm) pasted on a standard microscopic glass slide and with a coverslip to seal from the top. The gel concentration for most of the studies presented in this work is 1.5%. The effect of substrate properties was incorporated by varying the gel concentration in the range 1–2%. Growing colonies were observed in phase-contrast mode using Olympus IX83 microscope with a 60× oil objective (camera Hamamatsu ORCA-Flash, Japan). The whole system was set inside a thermally insulated temperature-controlled incubator (PeCon GmbH, Erbach, Germany). First, we located the coordinates of isolated cells on the agarose pad and the microscope was automated to capture these prerecorded coordinates and record the images of the colonies as they evolved (for the case merging of two colonies, two nearby cells were located and images were recorded as cells grew and divided forming colonies that eventually merged). The images are taken at regular intervals (typically 3 min). Analysis of phase-contrast images was performed using the open-source softwares Fiji:ImageJ (29) and Ilastik (30) and custom-written Python codes.

### Image segmentation and label-free tracking

The phase-contrast raw images were preprocessed by adjusting brightness and subtracting background noise using ImageJ. Then further background filling was done by applying top-hat and black-hat filter using Python-OpenCV. A number of these preprocessed images were trained in Ilastik by pixel classification of cells and background. The trained classifier was then applied to rest of the frames by using batch processing in Ilastik. This training step involved several iterations until high order of segmentation was achieved. The segmentation was done for frames until the colony attained MTMT, which was carefully noted manually by checking the pixel intensity of the images (Fig. S1, *A–D*). For the segmented cells, length (denoted $l_{c}$) was obtained by calculating the distance between the cell centroid and the center of two poles (which are two extreme ends of the contour of a cell). Cell width ($w_{c}$) was obtained using length and area of cell contour ($a_{c}$) using $w_{c}\approx \frac{4a_{c}}{\pi l_{c}}$. The length and width values thus obtained were further confirmed matching with extracted values of the major and minor axes length of each cell contour using the openCV ellipse fitting. The ellipse fitting also helped us to extract average orientation angle of cells. The colony outer boundary was extracted by image dilation and filling, which was used to extract the outer boundary perimeter and effective colony area.

**Figure 1:**
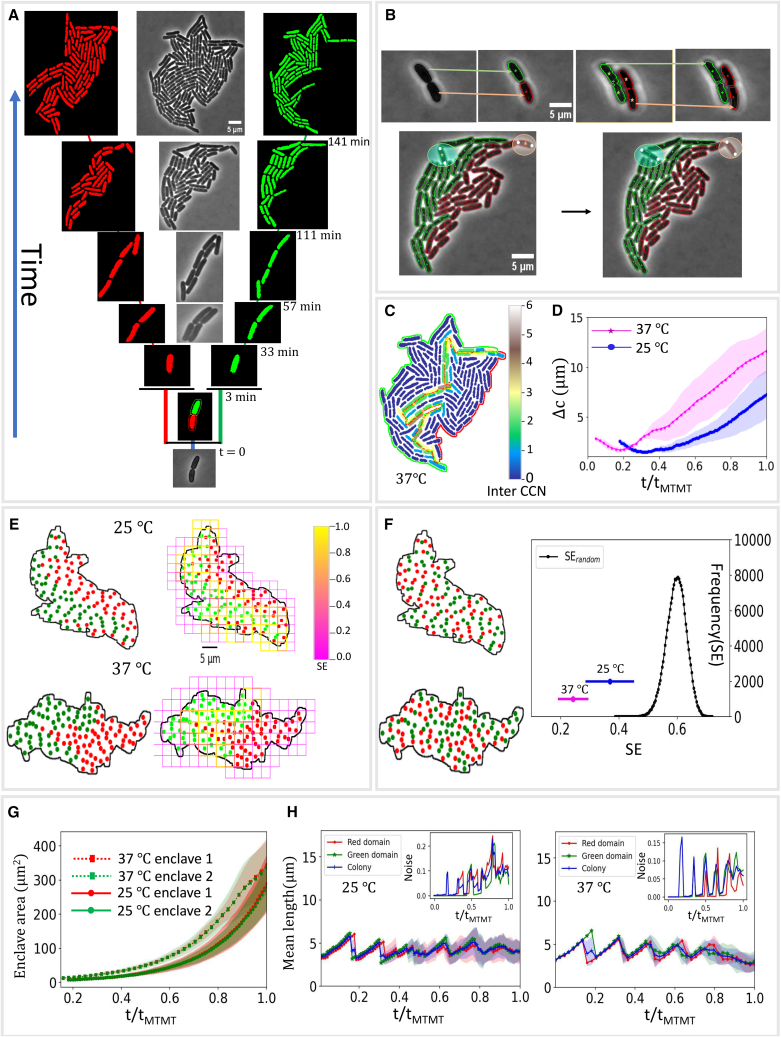
Emergence of self-similar enclaves in bacterial colonies. (*A*) Label-free tracking of a bacterial colony reveals partitioning of the colony into lineage enclaves starting from the initial two cells after the division of the founder cell. (*B*) Label-free tracking algorithm relies on frame-to-frame mapping of the centroid of cells to track them as they grow, with additional parameters based on mapping the cell poles and approximate prediction of daughter cell centroids to identify left out cells and division events. (*C*) Cell contact number (CCN) analysis to calculate neighbors of cells belonging to different progeny chain reveals that most of the cells only have spatial neighbors belonging to their own progeny chain. (*D*) The relative distance (Δc) between the centroids of the two enclaves is mapped as a function of time, where $t_{MTMT}$ denotes the time at which MTMT occurs. The error (SD) is shown by the shaded region in the plot. Here, $t_{MTMT}$ for colonies growing at 25°C is ∼332±12 min and for colonies growing at 37°C is ∼145±4.5 min. (*E*) Box-counting method employed to calculate Shannon entropy (SE) of cell arrangement patterns in colonies. (*F*) The ordered nature of enclave arrangement pattern vis-a-vis random arrangement patterns where progeny chain membership is assigned randomly to cells in the colony, fixing colony geometry and proportion of cells in each domain, two illustrative cases are depicted on the left. On the right, the black curve maps the values of SE for random arrangement patterns of cells in colonies, while the SE values for enclave arrangements for cells growing at 25 and 37°C are marked in blue and magenta, respectively. (*G*) The area of the two enclaves for colonies growing as a function of normalized time. (*H*) Mean cell length of cells in each of the two enclaves (*red* and *green*) as well as the whole colony (*blue*) is mapped as a function of normalized time for cells grown at 25 and 37°C. Inset: phenotypic noise for cell length (quantified by the normalized variance) is plotted for the two enclaves (*red* and *green*) and the colony (*blue*) as a function of normalized time.

Cell progeny chains were spatially mapped using a custom-built tracking code written using Python. To do that, first we extracted the features of the cells in all frames—centroid positions ($x_{c}$, $y_{c}$), length ($l_{c}$), width ($w_{c}$), and a label ($c_{k}$) were assigned to all the cells. We compare two consecutive frames (here referred as frames *t* and t+1) by looking for the “only” cell in frame t+1, whose centroid is within a cutoff distance d(c) from the centroid of a cell in frame *t* (initial t=0 is the time when the first pair of daughter cells are born). This pair of cells whose centroid displacement is within the cutoff were assigned to the same progeny chain. The cutoff value is appropriately chosen and optimized (around ∼0.75μm, which is of similar order as cell width). This covers most of the growing cells, but a few cells may be left out in frame *t* (either the cell in frame *t* has divided into two cells in frame t+1 or have undergone substantial movement compared with the cutoff distance). To track the cell that divides in t+1, we introduced “dummy” centroids for cells in frame *t*. Dummy centroids are points between the poles and original cell centroid, chosen by anticipating the centroids of the two daughter cells if the cell were to divide in the next frame. This helped us to map the dividing cell in frame *t* to daughter cells in frame *t* + 1. A small number of cells may remain after this, which may have undergone substantial movement (especially cells located near colony boundary which have free space to move). These were mapped by noting the displacement of their poles and the centroids within a cutoff distance in the two consecutive frames. If necessary, manual correction was done to rectify identification errors. Thus, we can spatially and temporally track the progeny chains emanating from the first two daughter cells (Figs. S1 *E*, *F*, and S2).

### Cell contact number analysis

We compute the average cell contacts (neighbors) of cells in the colony. For each cell in a given time frame, we first computed the distance between its centroid and centroid of other cells, and we collected the cells that were within cutoff radius ∼10 *μ*m from the original cell. Now, for these cells, we first selected a large number of points on their boundary and calculated the distance from points on the boundary of the original cell. If the minimum distance between two cell boundaries was less than a small tolerance value (∼1.2μm), then we called them neighbors of the original cell. The neighbors were matched manually in several instances to ensure correctness (larger tolerances allowed next neighbors to be identified as neighbors while smaller tolerances sometimes did not identify neighbors as neighbors). After this, we then looked at the fraction of inter- and intracontacts (at the level of progeny chains) based on the descendants of the first two daughter cells.

### Global properties of the enclaves

We now discuss how the centroid, perimeter, common interface length, and area of enclaves formed by progeny chains of cells were calculated. First we extracted the arrangement of the cells in the two enclaves based on the cell label information we get from the tracking algorithm and, thus, the outline of their outer boundaries. Then, we computed their centroid coordinates using OpenCV (the calculations were done frame by frame).

To estimate the common interface length of the two subcolonies, we first computed the perimeters of the outer boundary of the two enclaves formed by descendant cells, denoted $P_{s1}$ and $P_{s2}$. Since in all the cases the two enclaves are completely in the interior of the colony with common interface boundary, the interface length is simply given by

$$L_{interface}=\frac{P_{s1}+P_{s2}-L_{colony}}{2}$$

where $L_{colony}$ is the perimeter of the of the entire colony. Again the calculation is carried out frame to frame to track their temporal evolution.

### Shannon entropy for cell arrangement patterns in colonies

We leverage the concept of Shannon entropy (SE) to compare and contrast disparate cell arrangement patterns in growing colonies, which we describe now. We employed a moving box algorithm (box size, *s* ≈ 5.5 *μ*m) with box moved progressively by distance s/2 to run through the cells in the colony. Cells of the two enclaves having been assigned colors green and red and represented by their centroids, we calculated the probability of seeing a red cell or green cell in each box. Thus, for every box *B*,

$$SE\left(B\right)=-p_{r}\;\log\left(p_{r}\right)-p_{g}\;\log\left(p_{g}\right)$$

gives the SE of the arrangement in the box itself, where $p_{r}$ and $p_{g}$ are probability of finding a red and green cell, respectively. The average SE for each colony was then calculated by averaging the entropy values obtained for each (moving) box. Next, to get a measure of the magnitude of the obtained entropy value in comparison with the entropy values obtained for all possible cell arrangement patterns, only fixing colony geometry and the precise number of cells in each domain, we assigned the two colors red and green in same proportion to cells in the colony at random and computed average SE. This calculation was repeated in each case for $2\times 10^{5}$ iterations, to derive the range of values of entropy values sweeping through the phase space of all possible conformations and so as to estimate and compare the range of entropy values arising out of real-life patterns.

### Geometric features of enclave interface

To quantify enclave invasion, we probed the curvature of the interfacial curve and other geometric properties of the invasion front. For computing the local curvature of the interface curve, we utilized the kappa plugin in Fiji (31). Next, we calculated the area of the invasion of one enclave into other by a “hull” filling process to determine the region of invasion. Specifically, the high-curvature regions which marked the beginning of the invasion front of one enclave into other were joined to demarcate a region of invasion (see Fig. S11). The mean invasion width for enclave invasion by calculated by measuring the width of the invasion fronts in several locations and taking their mean.

### Orientation field of cells and topological defect detection in the colony

For all phase-contrast images, we first measured the orientation by the structure tensor method. The structure tensor was computed by taking the outer product of the image intensity gradient vector with itself for each pixel, locally averaged within a given Gaussian window for a size chosen roughly one-quarter the size of single cell. To retrieve the local orientation stored in the structure tensor, eigenvalue analysis was performed. One of the eigenvectors of the structure tensor encodes the orientation value (Φ) at each pixel lying between −π/2 to +π/2 (Fig. S14 *A*).

Next, we computed the nematic order parameter,

$$S_{R}={\left\langle \sin\;2\Phi \right\rangle }_{R}^{2}+{\left\langle \cos\;2\Phi \right\rangle }_{R}^{2}$$

$$0\leq S_{R}\leq 1$$

Here, the spatial average is done within fixed-size square region *R* (with each square roughly containing three to four cells) and the moving grid algorithm with steps of at least one-third size of grid size was employed to cover the entire colony for averaging (Fig. S14 *B*). Only pixels that resided in the bacterial cells of the colony (white pixels) were taken into account to compute the local order parameter. This was done to get rid of the false orientations, especially at the pixels near the cell boundary regions. Then we located the positions of low values of the order parameter by considering the points where $S_{R}\leq 0.3$. Of these points, local minima of the nematic order parameter values were taken as candidates for topological defect cores. For these possible defect sites, automatic defect detection was done by calculating the topological charge (*q*) (32). Only two types of defects (charge +1/2 and −1/2) were found (Fig. S14 *C*). Further manual correction of the defects is done by checking position of defects between at least two consecutive frames.

### Lattice model of colony growth and enclave formation

We start with discrete square lattice with 25×25 sites, which is large enough to simulate colonies of the size that are of interest here. We assume that each lattice site can be occupied by a single bacteria and no site is allowed to overlap. The initial configuration is of a “red cell” that is placed at the center of the grid (daughter 1) and its sister a “green cell” (daughter 2) is randomly allotted a site in the Moore neighborhood of the red cell. The simulation is then set off from this configuration, with the initial two cells dividing and their daughter cells dividing and so on and so forth, giving rise to their progeny chains. Every cell, starting from the initial two cells and then upon the birth of any new cell, is assigned a division time chosen at random using the log normal distribution, with the mean and standard deviation fixed to be the same as that gleaned from our biological data for colonies growing at 25°C (48±13 min) and 37°C (21±6 min) (as shown in Fig. S17 *A*). Based on the assigned division times, at each time step, we arranged the cells in ascending order, with the topmost cell in the list selected for division (thus, in the simulation, each time step corresponds to the time when a cell in the colony divides). Following each cell division event, we let one daughter cell remain at the same site as its mother cell site while the other daughter cell is allocated a randomly chosen site from the unoccupied Moore neighborhood of the site of the mother cell. The new cells are then assigned division times as described and the order of the cells according to division times is updated. These steps are repeated and new daughter cells continue to fill up the unoccupied Moore neighborhood of mother cells. As the number of cells increase, a stage is reached when the Moore neighbor of interior cells is completely occupied. For this, we introduce a shoving algorithm to allocate space for the daughter cells, whereby a direction from the mother cell site joining it to one of the sites in its Moore neighborhood is chosen with the probability of that direction being picked being given a weight to ensure that directions for which fewer cells need to be shifted is preferred (thus, the weight for a direction is inversely proportional to the number of cells that lie in that direction that need to be shoved). The cells are shifted outward by one site in that direction (mathematically, this is referred to translation along the chosen direction), thus creating an unoccupied site in the vicinity of the mother cell where one of the daughter cells is then placed. This rule allows us to effectively mimic growing bacterial colonies where cells push and jostle with their neighbors to allocate space for themselves and their progeny (we also consider an alternative shoving algorithm where the direction is chosen at random with equal probability; however, this does not change our results qualitatively). The simulation is continued in this manner until the number of cells increases to values consistent with those obtained from our biological data. Interenclave contacts of cells in these simulated colonies is calculated by looking at the number of cells lying in the Moore neighborhood that belongs to the opposite enclave. SE is calculated by a box-counting method, with box sizes chosen to ensure similar number of cells as in the case of SE calculations performed on our biological data. Averaging was done by performing over 20 simulations for each case (Figs. S17, *E* and *F* and S18, *A* and *B*). Furthermore, we also consider the case where upon division cells are not placed in the immediate Moore neighborhood but are also allowed to be placed in the Moore neighborhood of the original cell (thus in the lattice lying two steps away) (Fig. S18, *C* and *D*). In this case as well, the division times were chosen at random using the log normal distribution, with the mean and standard deviation fixed to be the same as that of our biological data for colonies growing at 25 and 37°C, with all other simulation parameters the same as in the previous case. Finally, we also consider simulations where cells belonging to one progeny chain divide faster while cells belonging to the other progeny chain divide slower (Fig. S19).

## Results

### Emergence of genealogical enclaves in bacterial colonies

To discern the emergent spatial structure of the genealogical organization in growing bacterial colonies, we tracked and studied intermixing dynamics of descendants of individual cells in single founder colonies of surface-associated bacteria, specifically *E. coli*. For this, we developed a new label-free tracking algorithm to spatially trace progeny chains emanating from the two daughter cells arising from the first division event of the colony, that of the founder cell (Figs. 1 *A*, S1, and S2). Such tracking is typically carried out by experimental labeling-based methods, usually involving different species or combinations of wild- and mutant-type cells. Here, we carry this out in an entirely label-free manner, leveraging a tracking algorithm that effectively logs the positions of cells as they grow and recognizes division events, spatially mapping progeny chains starting from the initial two cells (Fig. 1 *B* and materials and methods), after segmentation of phase-contrast images using the machine learning tool Ilastik (30) (see also tracking algorithms such as the fluorescence microscopy-based Schnitzcells (33) and Supersegger (34), which often require large amounts of input for image processing and analysis and newer deep learning-based algorithms (35,36), which give alternative label-free tracking methods).

**Figure 2:**
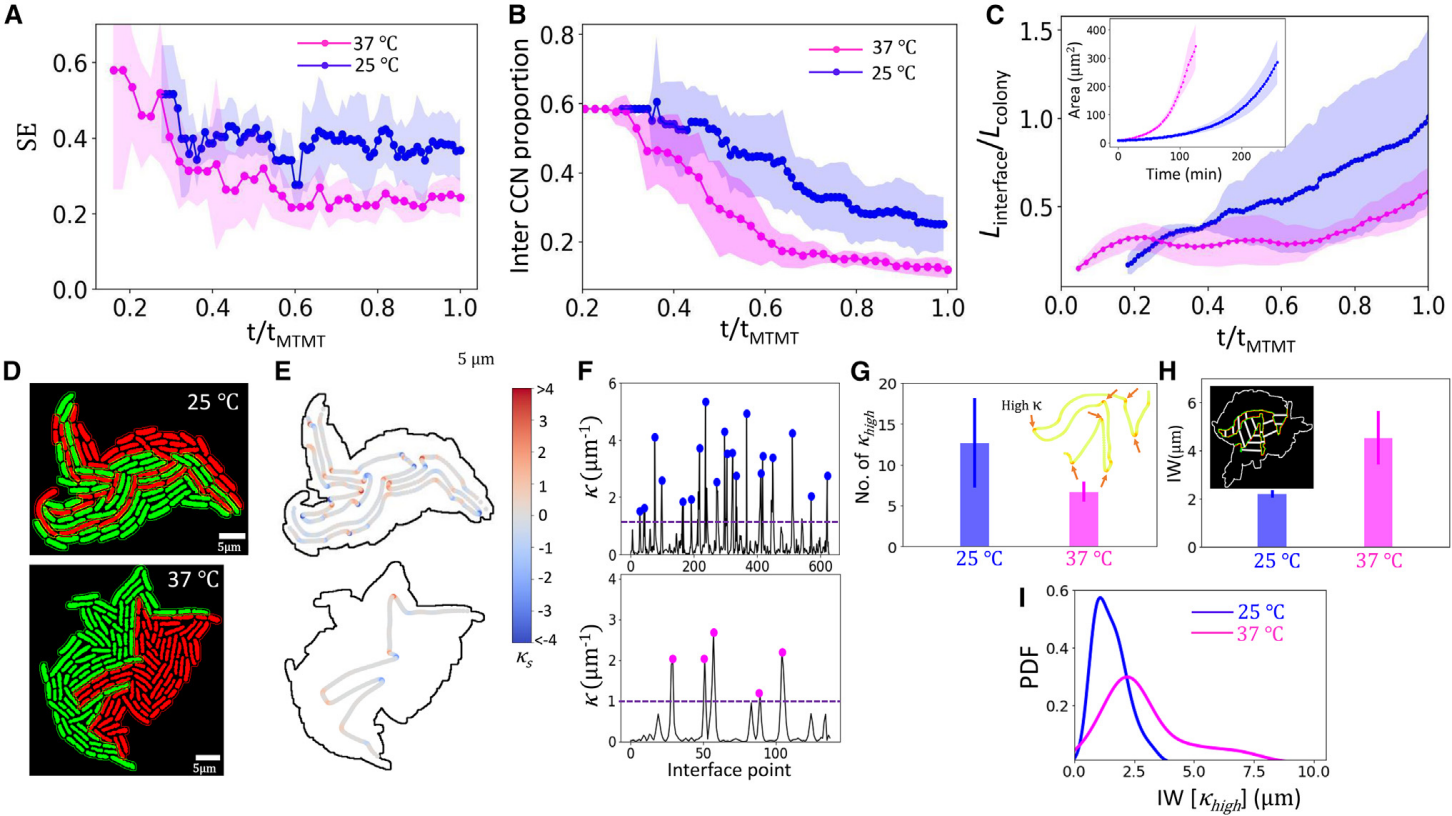
Activity governs dynamics of colony partitioning into genealogical enclaves. (*A*) Shannon entropy of cell arrangement patterns emerging from enclave partitioning of colony as a function of normalized time for cells growing at 25°C (*blue*) and 37°C (*magenta*). (*B*) Proportion of interenclave contacts of cells in the colony is plotted as a function of normalized time for cells growing at 25°C (*blue*) and 37°C (*magenta*). (*C*) The length of the interface between the two enclaves ($L_{interface}$) normalized by the perimeter of the entire colony ($L_{colony}$) is mapped as a function of normalized time for colony growing at 25°C (*blue*) and 37°C (*magenta*). Inset: the area of the two enclaves is mapped for colonies growing at 25°C (*blue*) and 37°C (*magenta*). (*D*) Genealogical enclaves in the colonies are shown (*red* and *green*) in two representative cases each for colonies growing at 25°C (*top*) and 37°C (*bottom*), with (*E*) the signed curvature ($\kappa_{s}$) of interfacial curves depicted in these two representative cases and (*F*) (modulus of) curvature (κ) values are mapped parametrized by the interfacial curve, with peak values of curvature above a threshold value (*dotted line*) highlighted by blue dots (for cells growing at 25°C) and magenta dots (cells growing at 37°C). (*G*) Mean ± SD of the frequency of high-curvature ($\kappa_{high}$) points in the interfacial curves is shown as a function of temperature. (*H*) Mean invasion width (IW) is plotted in the two cases of colonies growing at 25°C (*blue*) and 37°C (*magenta*). (*I*) Probability distribution (PDF) of invasion width in the vicinity of high-curvature regions (IW[$\kappa_{high}$]) of the interfacial curve.

For this study, we limit our discussions to monolayer configurations of bacterial colonies, i.e., before the colony undergoes mono- to multilayer transition (MTMT) (37). The cells of the two progeny chains undergo spatial intermixing as the colony grows, resulting in dynamic partitioning of the colony into two domains. Since the colony grows freely on the substrate, a certain degree of intermixing of lineages can be expected, which is also is seemingly entropically more favorable. However, we observed that cells from the two domains arranged themselves into enclaves, maintaining a spatial affinity for their close kin (Figs. 1 *A* and S2), having cell arrangement patterns very similar to that of merging of colonies, in which case it is more plausible and indeed observed that the cells from the two initial colonies form enclaves within the merged larger colony (Fig. S3). This is further confirmed by contact number analysis of cells in the colony (materials and methods), which shows that a large majority of cells in the colony have very few contacts with cells not in their progeny chain and, in fact, that such contacts decrease as the colony matures (Figs. 1 *C*, 2 *B*, and S4), consistent with the formation and consolidation of genealogical enclaves within the colony. Two interesting features of enclave formation that we observe additionally (we discuss them in more detail below), are 1) absence of “enclosed” enclaves, i.e., cells of one lineage completely surrounded completely by the cells of the other lineage (Fig. S10) and 2) the enclave formation is not evident during the very early stages of the colony formation (after just a couple of division events [Fig. S12]), independent of activity levels, suggesting that enclave formation and consolidation occurs at intermediate spatial and temporal scales relative to the timescales of colony development.

To quantify the relative spatial distribution of cells belonging to the two progeny chains, we tracked the evolution of the distance between centroid of the two enclaves (materials and methods). While in the case where cells from the two progeny chains were well intermixed, the centroids would have been close to each other, we observed the opposite, that the two centroids moved farther and farther away with time (Fig. 1 *D*). Thus, we conclusively infer that cells in progeny chains emanating from the first two daughter cells preferentially arrange themselves into enclaves within the colony. We also observe similar formation of progeny enclaves when we varied substrate properties by varying agarose concentration (Fig. S5) as well as for *V. cholerae* colonies, with cells having comparatively low aspect ratio (Fig. S6), highlighting the robustness of these phenomena under different conditions and species of bacteria.

Such dynamic partitioning of colonies gives rise to characteristic cell arrangement patterns. To compare the level of intermixing of these patterns vis-a-vis randomly intermixed distributions, we calculated the SE (38) of cell arrangement patterns for bacterial colonies (Fig. 1 *E*) and compared it to SE values obtained in case of randomly intermixed cell arrangement patterns, while fixing the colony geometry in terms of the relative positions of the cells as well as the proportion of cells belonging to both the lineages (Fig. 1 *F*, *left* shows two such colonies, see materials and methods for more details). For this, we use a moving box algorithm, which locally calculates the SE by determining the probability of a cell belonging to either of the lineages in the box and then averaging over the entire colony, thus capturing the intermixing levels at a granular level (as detailed in materials and methods). As the formula for SE suggests, predominant presence of cells of only one of the lineages, indicative of low order of intermixing, results in low values of SE while roughly equal presence of cells, indicative of high degree of intermixing, from both the lineages will result in higher values of SE. Thus, SE acts as an effective quantifier of the level of mixing displayed by the mosaic of patterns obtained when cells are assigned colors based on their ancestral line or otherwise, with high values suggesting a high order of intermixing and low values suggesting more ordered distributions. We observed that the SE is much lower compared with the case of randomly intermixed patterns (Fig. 1 *F*, *right*), underscoring the ordered nature of the spatial distribution of cells in bacterial colonies. Thus, cell arrangement patterns in bacterial colonies display much higher level of demixing compared with the average arrangement patterns when the colony spatial geometry is fixed, which also highlights that, in fact, natural cell arrangement patterns can potentially never attain average levels of intermixing. Notably, low values of the SE metric as well as the decreasing nature of its values as colonies grow (Fig. 2 *A*) indicate a propensity for ordering and self-organization as time evolves. This is analogous to the ubiquity of low thermodynamic entropy (indeed, thermodynamic entropy has the same expression as that of the SE when all microstates are equiprobable) and entropy reduction in myriad real-life patterns and situations (39,40), a hallmark of life itself (41). Notably, the SE metric for cell arrangement gives an effective way to distinguish spatial and temporal landscapes of biological systems, as has been applied sometimes for landscape studies in ecology (42).

We next probed whether cells in one of the enclaves displayed relatively better growth prospects, due to the changes in topology under the free expansion of the colonies on substrates, by measuring the difference in phenotypic traits of cells in the two domains. Firstly, we observed that the areas of the domains are approximately the same, around half the colony area (Fig. 1 *G*). The area comprises cells as well as intercellular voids, pointing to a striking similarity in the way cells accommodate and adjust spatially in the two enclaves. Further, mean cellular length, mean cell elongation rate, and average area of cells in the two domains show very close similarity with the colony-level statistics of the same (Figs. 1 *H*, S7, and S8, respectively). Next, to compare colony-level division statistics with that of the two domains, we calculated the number of division events occurring in the colony and the domains as a function of time. While at the single-cell level, this is a highly stochastic event (Fig. S17 *A*), we observed that the colony-level statistics show close proximity to the statistics of the two domains, as time progresses (Fig. S9). This reinforces that the two domains are a self-similar partition of the colony, transcending the dynamic nature of such partitioning. Furthermore, we observed that, in all cases, the two domains retained significant exposure to the surroundings of the colony and no “encircled” enclaves (i.e., subenclaves of cells from one progeny chain completely surrounded by cells from the other progeny chain) were formed as the colony grew. Indeed, the enclave perimeter exposed to outer surroundings is consistently around half the colony perimeter for both the enclaves (Fig. S10). This is important since boundary exposure is a crucial marker of survival fitness as it assures proximity to nutrients and space for expansion (14). Thus, despite growing freely in two dimensions and displaying a dynamically evolving colony geometry, the two domains originating from progeny chains of the two initial daughter cells gain very equitable access to growth resources and, resultantly, display very similar features as the colony at large.

### Activity governs dynamics of genealogical partitioning of bacterial colonies

A noticeable feature of bacterial growth is the dependence of biological activity (in other words, the rate at which bacteria assimilate nutrients to grow and divide, which can be observed by their growth rate) on factors such as ambient temperature, with cells growing and multiplying faster at optimal temperatures. While temperature has been used to tune activity in this work, other factors including nutrient availability, pH, and antibiotics can as well be used to modulate activity of expanding bacterial colonies. This difference in growth rates is quite apparent at colony scale but effect of activity on spatial geometry of the colony is completely obscured in standard imaging of cells, highlighting the importance of label-free tracking of cells. We observed an interesting effect of activity, which we modulate by varying the ambient temperature, on spatial geometry of colony—at slower growth rates, the colony displayed more intermixed structure, while at higher growth rates, the colony is more ordered and less intermixed. The time series of SE show that slower-growing colonies consistently display higher entropy of arrangement patterns (Fig. 2 *A*). Similarly, the distance between centroids of the two enclaves is lower while the proportion of interenclave contacts is higher for slower-growing colonies (Figs. 1 *D* and 2 *B*), confirming their higher degree of intermixing and less ordered features. We also observed a marked preference for narrow front invasion in this case, with thin fingers of cells invading into the region of the other, while for faster-growing colonies, wide front invasion is seen with the front comprising several cells. To quantify this difference, we study the geometry of interfacial region of the two domains (materials and methods). We compute the interfacial length normalized by the colony perimeter, observing that it is larger for the case of slow-growing colonies (Fig. 2 *C*). Further, high-curvature regions along the interface are comparatively more numerous for slow-growing colonies (Fig. 2, *D–G*), the presence of such regions attesting to the meandering nature of the interface and the affinity for narrow front invasion, which is characterized by sharp turns and, consequently, high-curvature hotspots along the interface curve (Fig. 2 *E*) (analogous to the case of river streams in hilly regions, characterized by tight bends and meandering curves compared with straight wide river streams in the plains). To quantify the size of the invasion front, we computed the mean invasion width, precisely identifying the invasion area (materials and methods and Fig. S11). We observed that mean invasion width is smaller for slow-growing colonies, highlighting their preference for narrow front invasion (Fig. 2 *H*). Finally, to understand the nature of invasion front in the vicinity of high-curvature regions, we calculated the probability distribution of invasion width in high-curvature regions, observing that for slower-growing colonies, peak is attained at low values of width while faster-growing colonies display a higher peak (Fig. 2 *I*), reiterating their propensity for narrow and wide front invasion, respectively. Therefore, we conclude that biological activity modulates the emergent spatial geometry of intermixing of cells in colonies, with higher activity resulting in more ordered, less intermixed colonies while lower activity promoting higher degree of intermixing, characterized by narrow front invasion of fingers of cells into the territory of the complementary enclave. It is notable that, at initial times after just a couple of division events, the colonies all displayed similar arrangement of cells but evolve with time into disparate arrangement patterns (Fig. S12). Thus, the dependence on activity level of cells on the arrangement patterns acts out after the colony has grown beyond a threshold size, reflecting the intermediate scale at which the phenomena take effect. This is further highlighted by the feature of such colonies to self-organize into a tapestry of small microdomains of similarly aligned cells, but with the size of such microdomains decreasing with increasing activity (12). Interestingly, *V. cholerae* cells which were grown at ∼25°C showed remarkable similarity of values of SE, centroid displacement, and normalized interfacial length with colonies of slow-growing *E. coli* cells (Fig. S13, *B*–*D*), implying that the dependence of spatial arrangement of cells on temperature-mediated activity is robust across cell shape and species.

### Genealogical enclave interface as hotspot of orientational disorder

**Figure 3:**
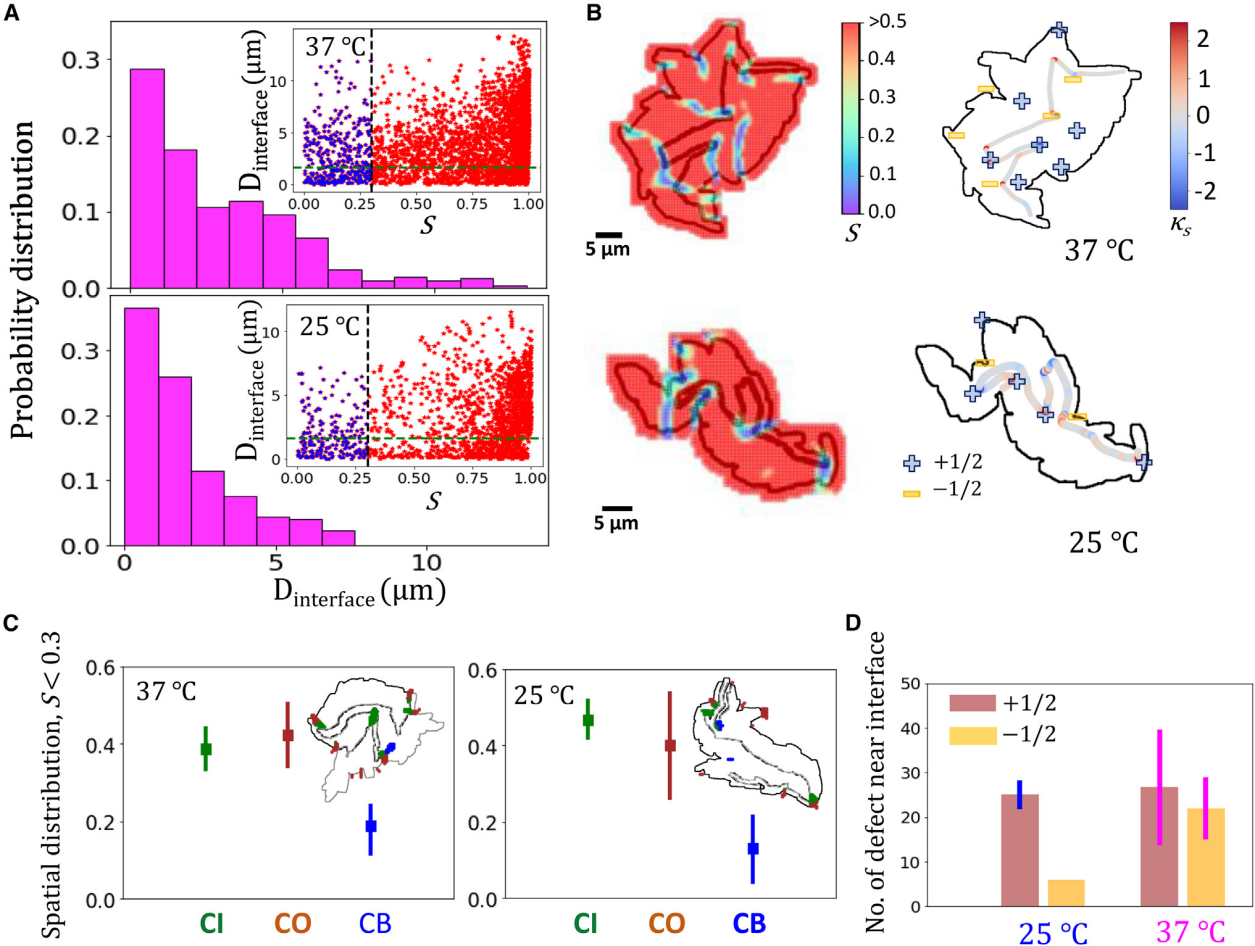
Low orientational order around enclave interface drives activity-dependent genealogical invasion. (*A*) Probability distribution of low nematic order parameter (S<0.3) regions as a function of distance from the interface for colonies growing at 37°C (*top*) and 25°C (*bottom*). Inset: for a representative colony growing at 37°C (respectively, 25°C), scatter plot of distance of grid points from the enclave interface is plotted as a function of nematic order parameter. The vertical dotted line shows chosen threshold value of low-order parameter (S∼0.3) and the horizontal dotted line shows the distance ($d_{i}$) from the interface where most of the low-order parameter points are lying ($d_{i}∼$ 1.5 *μ*m). (*B*) Color map of distribution of values of nematic order parameter *S* in shown for a representative case for colony at 37°C (*top left*) and 25°C (*bottom left*) and corresponding distribution of +1/2 and −1/2 defects are shown for the two cases: 37°C (*top right*) and 25°C (*bottom right*). (*C*) Spatial distribution of low *S* points (plausible sites for topological defects) that are located in the vicinity of enclave interface (denoted CI), near colony boundary (denoted CO) and in the rest of colony bulk region (denoted CB) for colonies growing at 37°C (*left*) and 25°C (*right*). (*D*) Distribution of +1/2 and −1/2 defects in the vicinity of enclave interface is shown for colonies growing at 25 and 37°C.

We seek to understand the mechanics of enclave invasion and its effect on orientational order of cells. Local orientational order of cells in colonies of nonmotile bacterial species has been shown to emerge from interaction of steric forces of cells shoving each other for space on one hand and active extensile stresses due to cell growth on the other hand, with the colony behaving like an active nematic liquid crystal (12,13). We hypothesized that enclave invasion will necessarily involve a high degree of jostling of cells, with the interfacial region emerging as a hotspot of orientational disorder. To investigate this, we compute the orientation order parameter *S* (materials and methods), a metric encapsulating local order in aligned systems, with S=1 denoting perfect alignment, while low *S* values signify a high degree of orientational disorder (43). We observe that regions of low *S* values tend to be along colony boundary and along the enclave interface (Fig. 3, *A* and *B*). Indeed, restricting to the colony interior, regions of orientational disorder occur almost exclusively along the enclave boundary (Fig. 3, *B* and *C*). We next tracked the location of topological defects in the colony vis-a-vis the spatiotemporal evolution of the enclave interface (materials and methods). Topological defects are singularities in local orientational field, associated with a breakdown of local orientational order and, thus, regions of *S* values are markers of the presence of defects (15). In the case of bacterial colonies, only +1/2 and −1/2 defects were observed (7,13). In our case, we observed that +1/2 and −1/2 defects proliferate in the vicinity of the enclave interface (Fig. 3 *B*). Grouping defects into those that are in the vicinity of the interface, those lying near the boundary, and those lying elsewhere, we observed that most defects are in the first two categories (Fig. 3 *C*). Furthermore, defects in the colony interior regions almost always arise in the vicinity of the interface (Figs. S14 *C* and S15). Thus, while regions of high orientational disorder and presence of defects in the interior of bacterial colonies may seem random, label-free cell tracking allows us unravel this riddle by exposing the active-active interface (44) of progeny enclaves as a hotspot for orientational disorder and topological defects. A small number of defects are observed to arise elsewhere in the colony. These defects may lie at the interfaces of subenclaves (comprising the lineages of the first two cells within an enclave, for instance) or, as has been suggested, at the boundary of microdomains of similarly aligned cells (12). But, as we show, the majority of defects in the colony bulk lie adjacent to the enclave interface, suggesting that the relatively large sizes of the constituent enclaves, compared with the subenclaves and microdomains, play a role in the defect formation close to the enclave interface. It is also pertinent to note that majority of the cells along the interface preferentially align parallel to the enclave interface (Fig. S14 *A*), as has been observed for interfacial regions in several other contexts (44,45,46). Nevertheless, this preference is broken into regions of high curvature of the interface curve and we observed that regions of high disorder in the orientational field as well as presence of defects correlated strongly with regions of high curvature along the interface (Fig. 3 *B*).

We also tracked the dynamics of defects vis-a-vis enclave invasion and observed that invasion is typically initiated by defects as one enclave pushes into the region of the other. Over time, such invasions developed while the initial defect persisted in the region, signifying the role of defects in nucleating and fostering invasion (Fig. S16). This is highlighted by looking at defect type distribution in enclave interface vicinity as a function of activity (Fig. 3 *D*), which shows that −1/2 defects manifest in higher numbers for faster-growing cells. Furthermore, the geometry of defects is closely related to the invasion mode fostered by them. +1/2 defects have a comet-like shape and behave like self-propelled particles (47), traveling comparatively larger distances on average (Fig. S14). This points to their tendency to form and develop narrow front invasions that penetrate deeper (Fig. S16). However, −1/2 defects have a trifold symmetry and display a propensity to be “caged” in that they move comparatively shorter distances, showing a saturating trend in their movement (Fig. S14 *E*). This attributes their tendency to nucleate and develop wide front invasion, hampered by their symmetry from penetrating deeper but slowly spreading over a larger region (Fig. S16). Such contrasting features of the movement of +1/2 and −1/2 defects have been noted before, for bacterial colonies (13) and other biological aggregations (48).

### Adhesion and stochasticity in cell division intervals mediate enclave evolution

**Figure 4:**
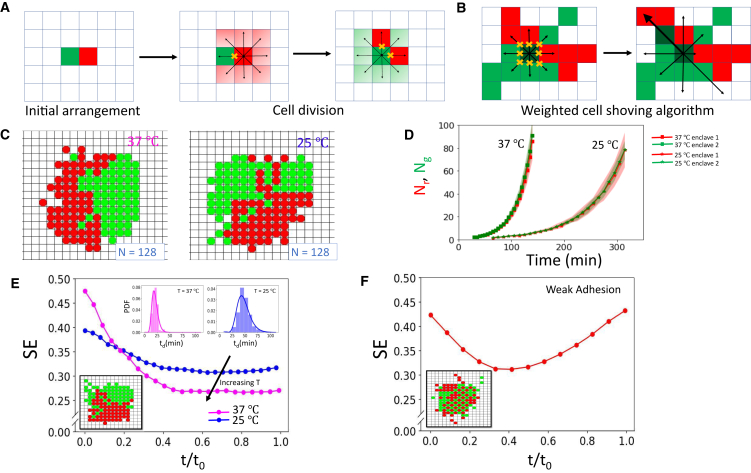
Lattice model simulations capture emergence of enclaves in qualitative agreement with experiments. (*A*) The simulation starts with two cells placed next to each other randomly. Division time for cells are assigned according to a log normal distribution whose parameters are determined from our biological data. Once the cells divide, one of the daughter cells occupies the original site while the next is assigned an empty site in its Moore neighborhood, assigned randomly with equal probability. (*B*) Once the Moore neighborhood is completely occupied, upon cell division, one of the daughter cell occupies the original site and the other “shoves” the cells in a direction chosen according to a probability distribution so as to ensure that cells prefer to shove in directions which have less number of cells that must move. (*C*) Lattice model simulations of colonies growing at 25 and 37°C are shown here for two representative cases with number of cells *N* = 128 and the two progeny chains colored red and green. (*D*) The number of cells in each domain is plotted as function of time. (*E*) Shannon entropy of arrangement pattern is plotted as a function of normalized time for lattice model simulations with high (*magenta*) and low width (*blue*) division time statistics to simulate colonies growing at 25 and 37°C, respectively. Inset: distribution of division times and snapshot of a simulated colony. (*F*) Evolution of Shannon entropy of arrangement pattern with normalized time for the case where a daughter cell is allowed to be placed away from the neighborhood of the mother cell (in particular two lattice steps away), thus not requiring two daughter cells to always be neighbors, in effect weakening the adhesion of daughter cells. Inset: snapshot of a simulated colony in this case.

To understand the physical principles underpinning enclave formation in bacterial colonies, we employed a lattice cell model. Lattice models have been extensively and fruitfully used to model cellular agglomerations and microbial colonies, including bacterial colonies (25,49). In our case, starting from a single cell, a point in a lattice, colonies were simulated to grow by a division process whereby a cell divided into two daughter cells with one of the cells occupying the lattice point corresponding to the mother cell and the other daughter cell occupying a neighboring cell, according to specified rules incorporating stochasticity and shoving, which is introduced to simulate the ways cells jostle and make space in crowded conditions in the colony bulk, reminiscent of jamming in colloids (50) and in microbial agglomerations of species such as yeast (51). Cell division statistics in our simulations followed a log normal distribution with parameters determined from our biological data (Figs. S17 *A*, 4 *D*, and S17 *C*; materials and methods). The cell agglomerations were then simulated to grow until they reach cell numbers at MTMT, consistent with our biological data. We observed that enclave formation was always well displayed by the simulated colonies, with very few isolated, encircled progeny domains and the two domains displaying very similar size to each other as they grow (Fig. 4, *C–E*), a qualitative agreement with experimental data.

We draw analogy from mixing-demixing in binary mixtures, where the free energy of the system is $F=E_{i}-TS^{mix}$ (52,53,54), with $E_{i}$ being the interaction energy and $S^{mix}$ the mixing entropy, which promotes mixing of the components. In our case, intermixing is driven up by the shoving of cells, which drives them farther from their genealogical milieu and promotes intermixing. However, as we can observe from colony growth images, sister cells remain in contact after division. This associates an energy cost to cell shoving, to separate a cell from its sister. To test this, we slightly weakened their tendency to remain in contact upon division (materials and methods). We observed that such a minor change is in fact sufficient to cause distinctly different behavior, in particular exhibiting high degree of intermixing and resulting in an increase in SE with time (Figs. 4 *F* and S18, *C* and *D*), even though viable colonies are still formed. This underscores the importance of adhesion of daughter cells upon division, likely due to self-recognizing adhesion molecules or mediated by EPS production (55,56), for the formation of genealogical enclaves. In general, intercellular adhesion can be shown to modulate colony morphology with very high levels of adhesion giving rise to chaining phenomena in colonies due to cells adhering at their poles after division (57).

Next, we wanted to probe the effect of self-similar growth of progeny chains, as displayed by bacterial colonies as well as our simulations (Figs. 1 *G* and 4 *D*), in promoting enclave formation. For this, we differentiated the division times of cells by slowing down the division time for cells belonging to one progeny chain. For small differences in division time, enclave formation is still prevalent (Fig. S19), underlining the robustness of enclave formation even when varying growth levels of cells results in a difference in the number of cells in the progeny chains as they grow (e.g., when wild-type cells and mutant cells having a small variation in growth rates are grown together). However, when the difference in growth rate is large, leading to larger imbalance between the number of cells in the progeny chains, we observe a high order of intermixing (Fig. S19), similar to the case of binary mixtures, where for low concentrations of one of the components, mixing is displayed as demixing becomes energetically prohibitive (54). This is analogous to the phenomena of clonal expansion, when a mixture of bacterial strains are grown together and one of the strains is handed a growth advantage (58,59). Interestingly, while our simulations run until the number of cells are similar to cell numbers at MTMT as in our biological data, we observe that differences in SE values (Fig. 4 *E*) and contact numbers (Fig. S17 *E*) are seen to become nearly constant. This suggests that these effects persist even when the system grows even larger in size, although eventually limitations in available space and nutrient availability, which we have not considered in our simulations, will come into play as colonies grow larger and might affect cellular organization in colonies.

Finally, our simulations also showed a dependence of intermixing on activity as our analysis of bacterial colonies had established. Specifically, we calculated the proportion of interenclave contacts of cells and the SE of colonies obtained from lattice model simulations and observed that both are consistently lower for faster-growing colonies (Figs. 4 *E* and S17 *E* and *F*, and compare with Fig. 2, *A* and *B*), suggesting a lower degree of intermixing in this case, as in the case of bacterial colonies. Here, activity is encoded by division times gleaned from our biological data, with less-active colonies having a wider spread of division times. Notably, the coefficient of variation in both cases are the same (∼0.27), agreeing with results in (60). Thus, lattice model simulations suggest that stochasticity in cell division times, engineered by biological activity, is a factor in determining the disorder in the emergent organization of cells. This is likely due to effective space grabbing by both progeny chains for faster-growing cells, where lesser spread in division times ensures that space is filled in orderly fashion with both progeny chains occupying sites one after the other. However, for slower-growing cells, the wide spread in division times can lead to cells from one progeny chain dividing multiple times before a cell from the other chain can divide, leading to differential site occupation, locally in space and time. Still, the large difference in SE values for cells in bacterial colonies suggests that there are other factors at play as well. For instance, elevated levels of intercellular adhesion can increase clonal mixing in large colonies (57). Furthermore, the characteristic patterns forming due to enclave invasion are reminiscent of mixing of viscous fluids (61,62), occurring when a low viscosity liquid displaces a high viscosity liquid under pressure. In the case of enclave invasion, however, there is no difference in material properties between the two enclaves and it is activity that drives the qualitatively similar invasion dynamics. However, for faster-growing cells, it is likely that cell-to-cell tensions are higher near the interface (63), which can increase effective viscosity for cell aggregates, resulting in blockage of deeper thin invasions and giving rise to wide, shallow invasion fronts with more ordered cell arrangement patterns as observed. A future study utilizing agent-based simulations and more elaborate lattice simulations, incorporating cell orientation, will be done to understand the role of cell-cell and cell-substrate interactions in activity dependence of intermixing dynamics in growing bacterial colonies.

## Discussion

Emergence of genealogical enclaves is a universal feature of sessile colonies, which is conserved across temperature differences and activity levels, substrate properties, and species, as revealed by our custom-built label-free tracking algorithm. The self-similarity of the enclaves on several key phenotypic traits suggests comparable growth prospect of the progeny cells, despite local biophysical differences including stochasticity in division times (7) and mechanoregulation of the cell growth due to heterogeneous packing (8). Self-similarity, which refers to the property of an object of being similar to a part of it, is a key feature of fractals and several interesting natural objects display self-similarity including branching in trees, vegetables such as broccoli, and coastlines across the globe. Such self-similarity is striking in the case of bacterial colonies, since stochasticity in phenotypic traits can get compounded as the colony grows to effect qualitatively different features in the two enclaves. Some features of genealogical demixing have been studied in earlier works (27,58,59,64), which typically deal with strains of bacteria mixed together in fixed proportions and grown to much larger size than our case. While the central region is mixed, clonal sectors of cells resulting from a demixing of strains in observed toward the edge of cell agglomerations in these studies. Size plays a role in the formation of such sectors since, as the agglomerations grow, the cells at the expanding front get much better access to nutrients, while cells trapped behind suffer nutrient depletion and waste product accumulation (65). On the other hand, our study starts with a single cell and the colonies are grown to much smaller sizes, until MTMT is attained, when nutrient availability is uniform. In particular, our study studies the genesis of genealogical enclave formation in bacterial colonies and shows that it occurs at very early stages of colony formation, thus hinting at its universal nature in microbial agglomerations.

Biological activity is a key determinant of microbial life, driving growth and propagation, and modulated by multiple factors such as temperature and nutrient availability. However, uncovering the effects of activity on spatial and genealogical organization of cells in colonies have remained largely unexplored. Our work highlights important differences in the way cells arrange themselves within colonies as a function of activity: slow-growing cells display higher degree of intermixing among progeny chains and a preference for narrow front enclave invasion, while faster-growing cells show less intermixing and a preference for wide front of enclave invasion. Such features remain completely hidden when standard imaging and analysis of colonies is done and become apparent only when we use lineage tracking and in depth quantitative analysis. In general, the methods developed here allow a comparison between cell organization patterns in diverse settings. Interestingly, somewhat similar phenomena have been observed when differently labeled *E. coli* cells are mixed and grown to much larger sizes, with the geometry of clonal sectors showing a difference as incubation temperature is changed: very few thick sectors are observed at ∼37°C while several thinner spoke-like sectors arise at ∼25°C (58).

The enclave interface emerges as a hotspot for orientational disorder, with regions of high disorder and appearance of topological defects in the colony interior predominantly occurring near the enclave interface. Activity dependence is discerned in this case as well, with faster-growing colonies displaying a relatively high proliferation of −1/2 defects in interface vicinity, whose trifold symmetry and suppressed mobility correlates with a typical wider front of enclave invasion observed in this case. The role of the enclave interface as an active-active interface, near which defect dynamics is an emerging field of study (44), is also underlined. Further studies in this direction, utilizing theoretical and simulation-based methods, such as phase field modeling, will shed more light on the morphodynamics of enclave interfaces and their relation to the transport properties of topological defects. Drawing analogy from the thermodynamics of binary mixtures and phase separation, our lattice simulations reveal the critical role of cell-cell interactions in determining the extent of lineage mixing. Further work is needed to derive the precise theoretical underpinnings of the phenomena of genealogical demixing and to generalize our methods to study colony growth beyond MTMT, thus leading to an understanding of lineage distributions in multilayered colonies. It will be particularly interesting to compare self-similarity inside layers and across layers, which will be in effect a display of multidimensional self-similarity. With bacteria commonly occurring in multispecies consortia, e.g., microbiomes associated with diverse biotic and abiotic settings, our results also propose potential mechanistic basis of the evolutionary benefits of symbiotic relations existing within multispecies systems. Beyond single species, this work shows a way in which bacteria in a multispecies community could benefit doubly: from the enclaves of similar cells on the one hand, and on the other hand by maintaining symbiotic relations with other species, thereby ensuring enhanced chances to fend off stressors. Looking forward, it would be valuable to track the growth of healthy versus stressed cells, representing diseased and healthy states, to discern differences in the way cells organize in such conditions and their evolution with time. While comparative studies on species diversity have been done in several cases (66,67), the emerging spatial patterns and their genealogical evolution remain lacking. The methods developed in our work can be utilized to answer these open and relevant questions, ultimately to elucidate the role of proximity to kith and kin in maximizing fitness and viability.

## Data and code availability

Data that support the plots and findings of this study are available from the corresponding author upon reasonable request.

## Acknowledgments

We gratefully acknowledge the support from the Institute for Advanced Studies, University of Luxembourg (AUDACITY grant: IAS-20/CAMEOS to A.S.) and a Human Frontier Science Program Cross Disciplinary Fellowship (LT 00230/2021-C to G.R.). We thank J. Nguyen for the raw image displayed in Fig. S6 *A*. A.S. thanks Luxembourg National Research Fund for the ATTRACT Investigator Grant (A17/MS/ 11572821/MBRACE) and a CORE Grant (C19/MS/13719464/TOPOFLUME/Sengupta) for supporting this work.

## Author contributions

Conceptualization, planning, administration, and supervision, A.S.; methodology, A.S. and G.R.; investigation, data and statistical analysis, computations and modeling, G.R. with inputs from A.S.; writing, G.R. and A.S.

## Declaration of interests

The authors do not declare any conflicts of interest.

## Supporting material

Document S1. Figures S1–S19

Document S2. Article plus supporting material
