# Supplementary material for: Growing bacterial colonies harness emergent genealogical demixing to regulate organizational entropy
Source: Biophys Rep (N Y). 2024 Aug 26;4(4):100175. doi: 10.1016/j.bpr.2024.100175 (PMC11416667; doi:10.1016/j.bpr.2024.100175)
Supplement: Document S1. Figures S1–S19 [file mmc1.pdf]

Biophysical Reports, Volume *4*

Supplemental information

Growing bacterial colonies harness emergent genealogical demixing to regulate organizational entropy

Garima Rani and Anupam Sengupta

Emergent genealogical demixing suppresses temporal entropy of cell arrangement in growing bacterial colonies

Garima Rani[1], Anupam Sengupta [1,2*]

[1]Physics of Living Matter Group, Department of Physics and Materials Science,
University of Luxembourg, 162 A, Avenue de la Faïencerie, L-1511, Luxembourg
[2]Institute for Advanced Studies, University of Luxembourg,
2, Avenue de l'Université, L-4365, Esch-sur-Alzette, Luxembourg
*E-mail: anupam.sengupta@uni.lu

Supplementary Information

This file contains:
Figures and captions for Supplementary Figures 1-19.

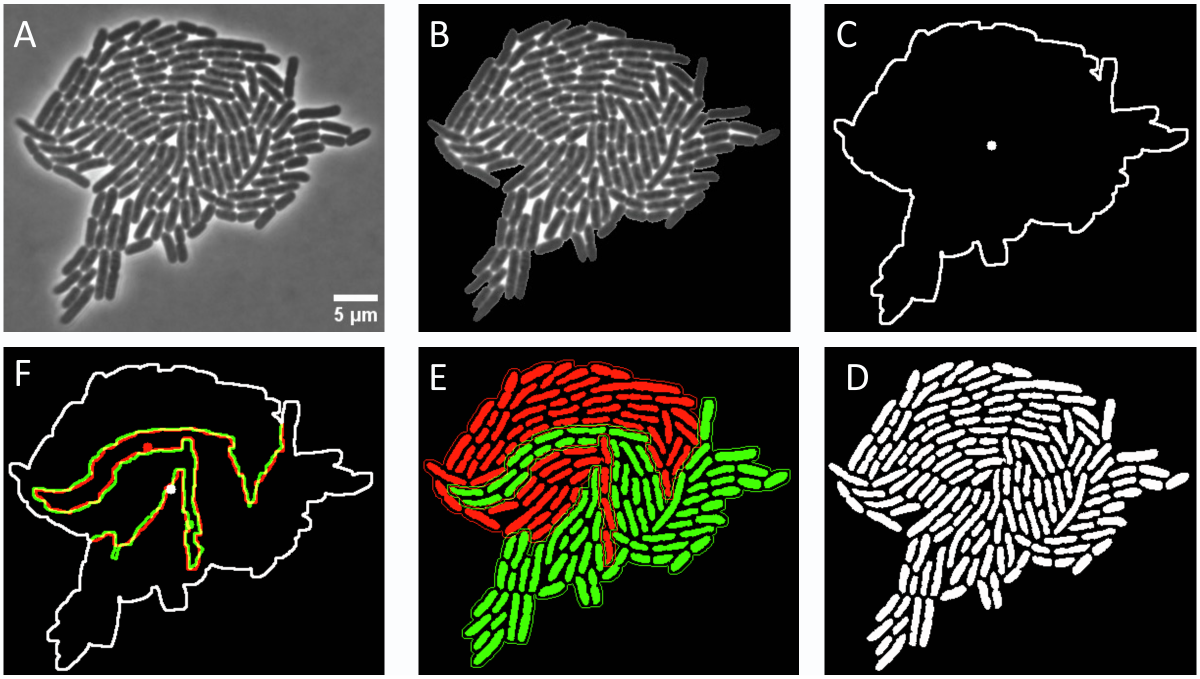

Supplementary Figure 1: Image analysis pipeline. **A.** Phase contrasted raw image, **B.** Image contrast is adjusted and background filling done applying top hat and black hat filter using Python-OpenCV. **C.** Colony boundary and centroid (white dot) is extracted by deducing the outer contour using image dilation and space filling. **D.** Image segmentation is done by training in Ilastik. **E.** Label-free tracking was used to deduce the two progeny chains(red and green color denote the two enclaves formed by the descendant cells). **F.** Enclave centroid (red and green dots) and colony centroid (white dot) are shown within the colony boundary with the interface of the two enclaves shown by red-green overlapping lines.

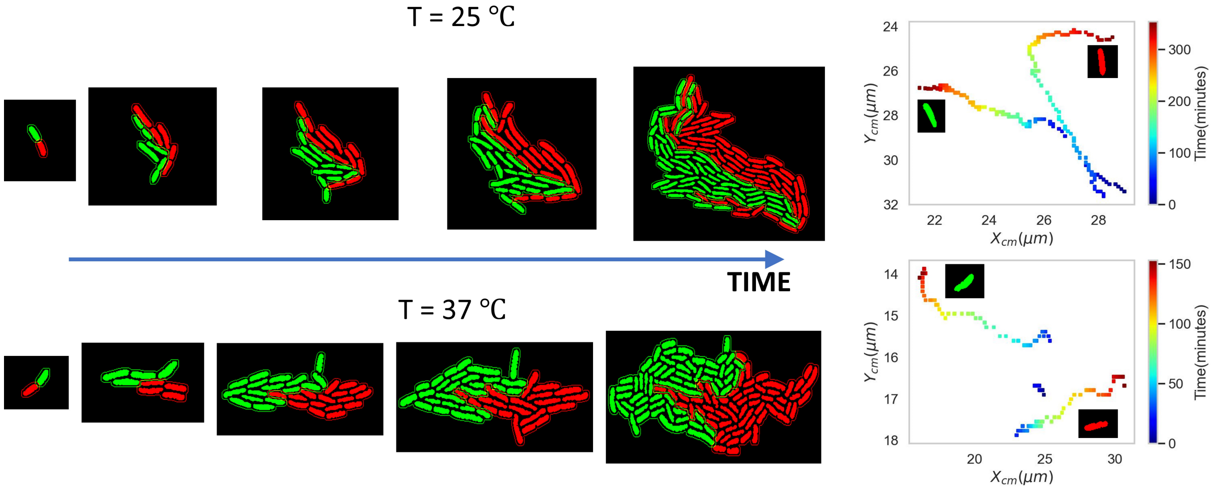

Supplementary Figure 2: Enclave formation and evolution- Spatiotemporal evolution of progeny enclaves is shown for representative colony growing at 25 °C (top) and 37 °C (bottom). On right, colormap of the position of the centroid of the two enclaves as they evolve with time is shown in each case.

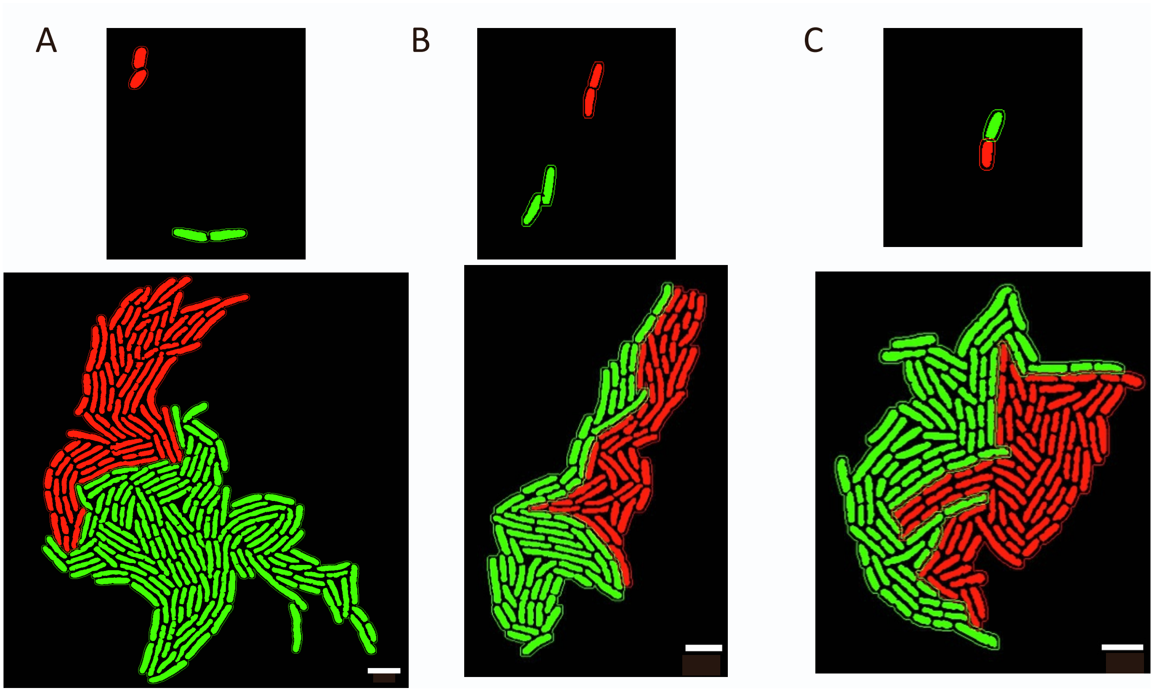

Supplementary Figure 3: Cell arrangement patterns as two colonies merge and as the progeny enclaves emerge in single colonies are analogous. Starting with two founder cells, the merging of two colonies (colored red and green) to form a larger colony is shown here in two instances (**A** and **B**). Notably, the two colonies upon merging show very similar spatial organisation of cells in terms of partitioning of cells into enclaves as the case of progeny enclaves originating from a single founder cell, shown here for a representative case (**C**). The scale bar (white line) represents 5 μm.

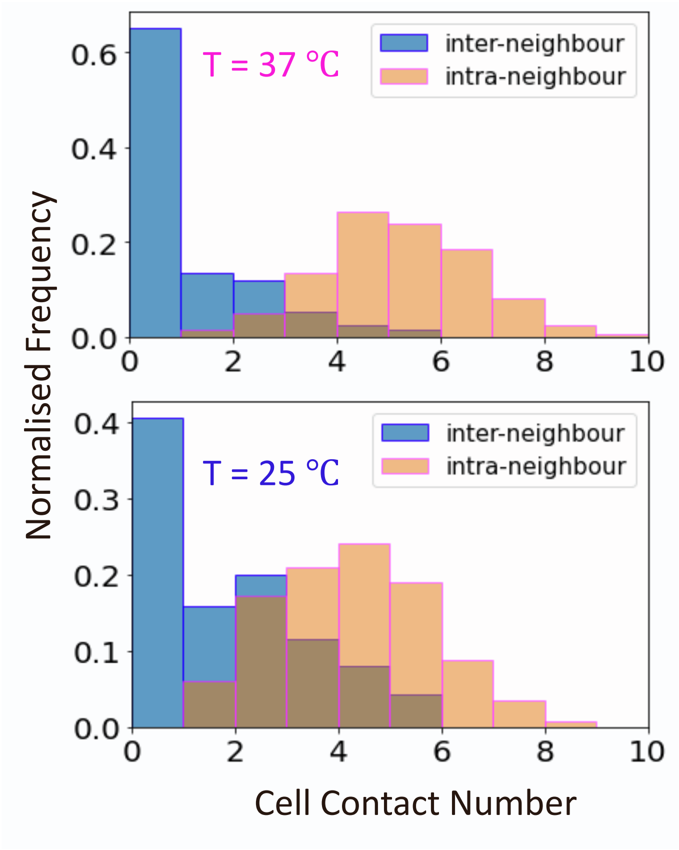

Supplementary Figure 4: Cell contact number distribution- Normalised frequency distribution of cell contacts with inter-enclave and intra-enclave contacts colored blue and orange, respectively.

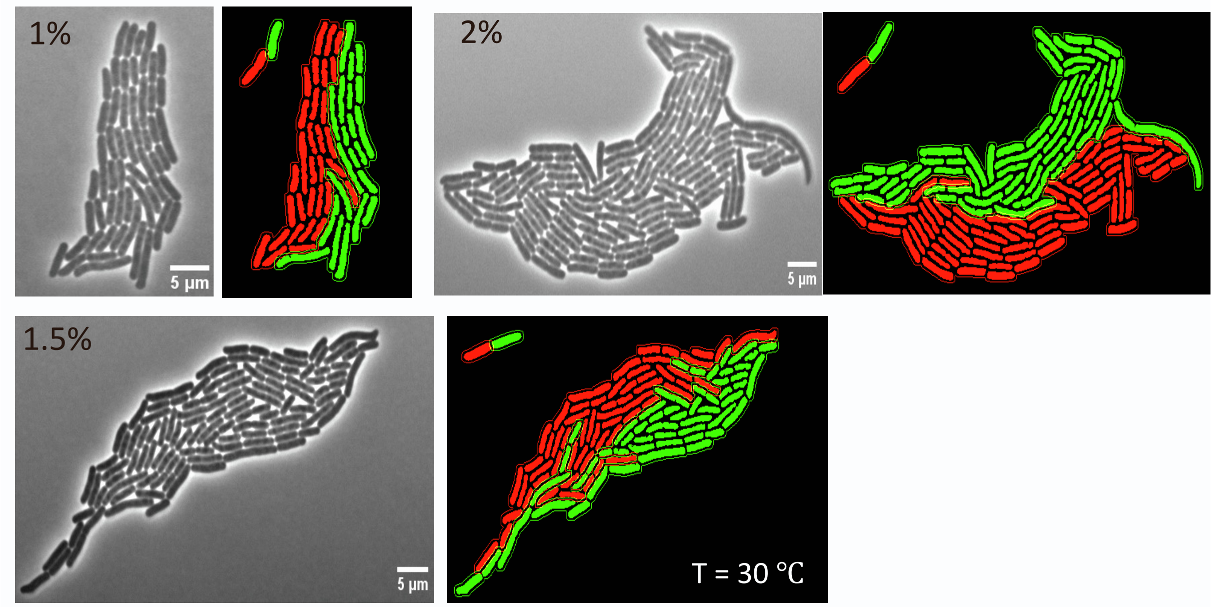

Supplementary Figure 5: Colonies grown on varied substrates also show formation of progeny enclaves. Colonies grown on substrates with varying agarose concentration (1%, 1.5% and 2%) also display formation of progeny enclaves. The inset in all cases shows the two daughter cells arising from the first division event of the colony.

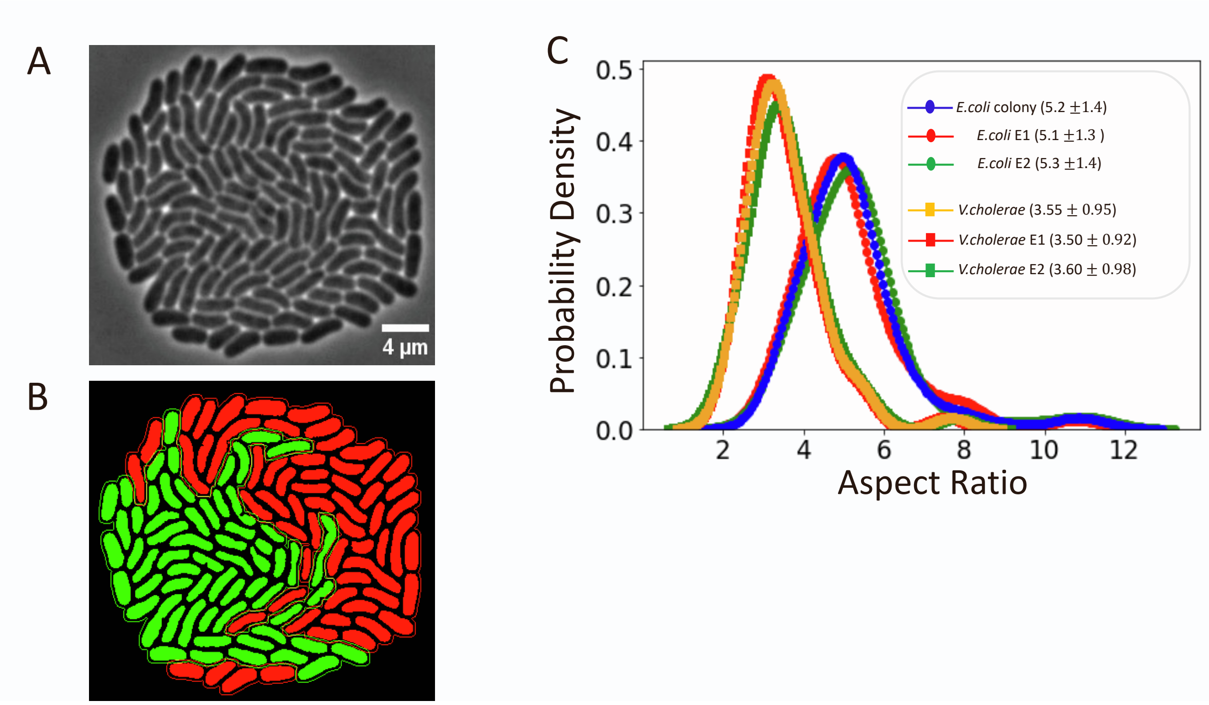

Supplementary Figure 6: Low aspect ratio cells also display enclave formation. Progeny enclaves is displayed is case of other bacterial species as well, specifically *V.cholerae* growing at 25 °C. **A.** Raw image of the colony at MTMT (Courtesy of J. Nguyen), **B.** The appearance of enclaves obtained using label-free tracking suggests that it is robust for non-motile species of bacteria independent of cell shape. **C.** Probability density of the aspect ratios of the cells for *V. cholerae* and *E.coli* considered in this work, depicted for the two enclaves as well as the whole colony. The *V.cholerae* cells clearly have low aspect ratio (henceforth referred to as LAR strain). The probability density of the cells in the two enclaves show very similar aspect ratios in both cases, an exibition of self-similarity of the two enclaves for both the species.

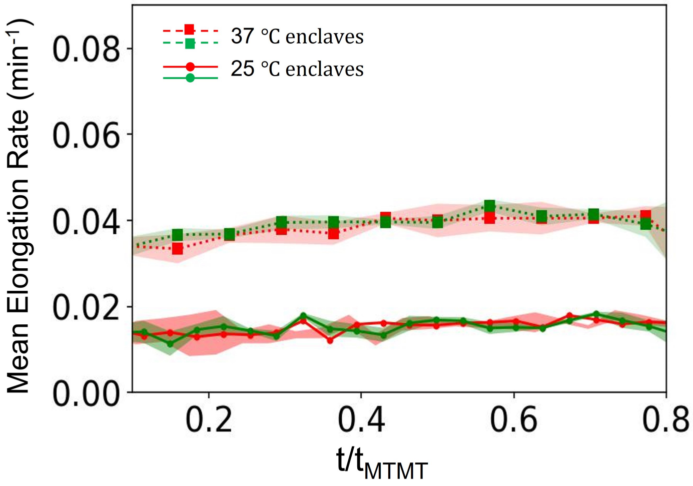

Supplementary Figure 7: Self similarity between the enclaves and the colony as they grow. Mean elongation rate of cells in the two progeny chains (colored in red and green for both cases) is plotted as a function of time for cells growing at 37 °C (dotted lines with squares) and at 25 °C (lines with circles). The mean elongation rates for the colonies are - 0.015 min^{-1} for colonies growing at 25 °C and 0.037 min^{-1} for colonies growing at 37 °C.

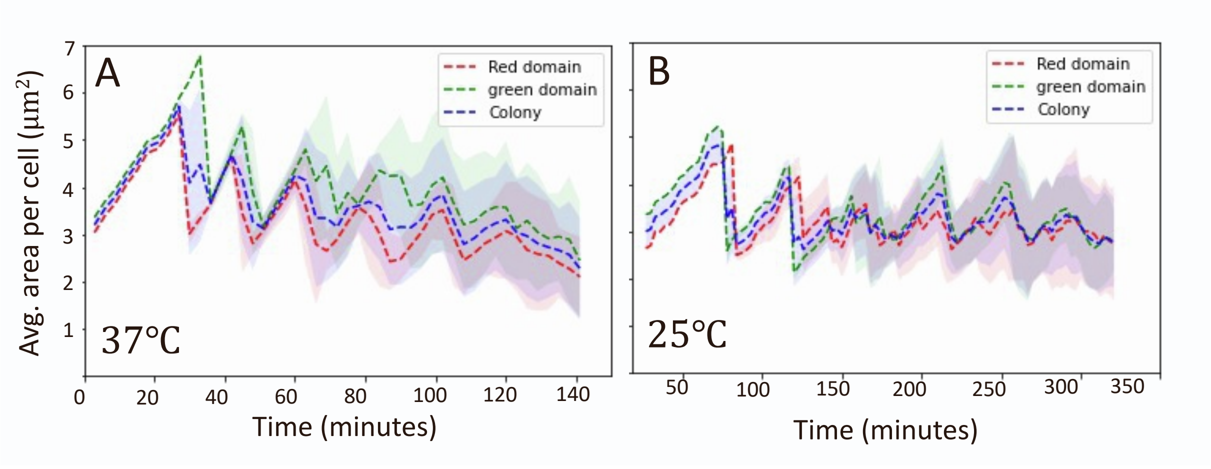

Supplementary Figure 8: Self similarity between enclaves and colony in the average area occupied by a cell. Average area of a cell in the two enclaves (colored red and green) and the colony (colored blue) is plotted as a function of time for cells growing at 37 °C (**A**) and at 25 °C (**B**).

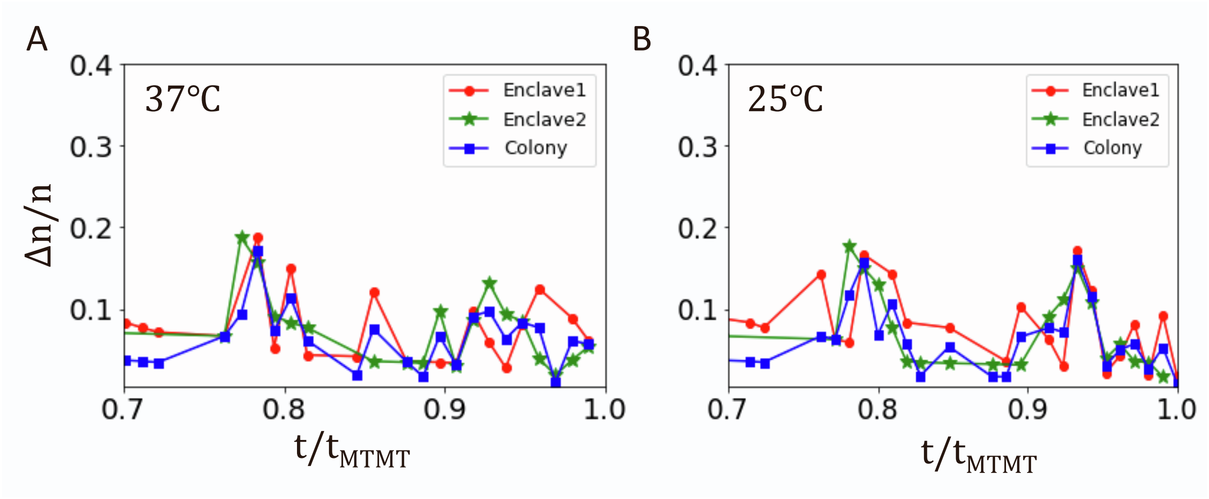

Supplementary Figure 9: Division events in the colony and the enclaves evolve similarly with time Normalised number of division events in the colony is plotted as a function of normalised time for the two progeny enclaves (colored red and green) and for the colony (blue) in case of cells growing at 37 °C (**A**) and 25 °C (**B**).

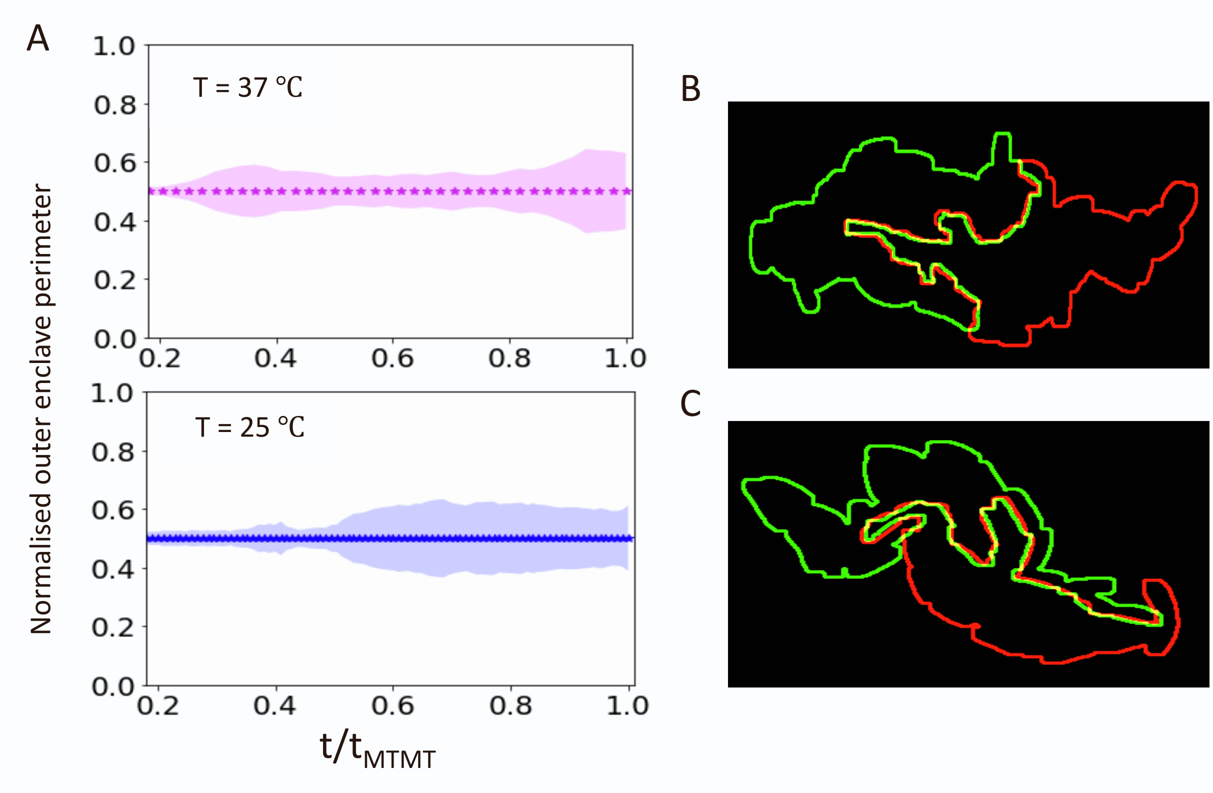

Supplementary Figure 10: Enclaves have identical exposure to external surroundings. A. Enclave perimeter which is exposed to the outer surroundings (referred to as outer enclave perimeter) for each enclave is normalised by colony perimeter and plotted as a function of dimensionless time (t/t_{MTMT}). **B,C.** show the enclave outer boundary and the enclave interface for a representative colony at 37 °C (**B**) and 25 °C (**C**).

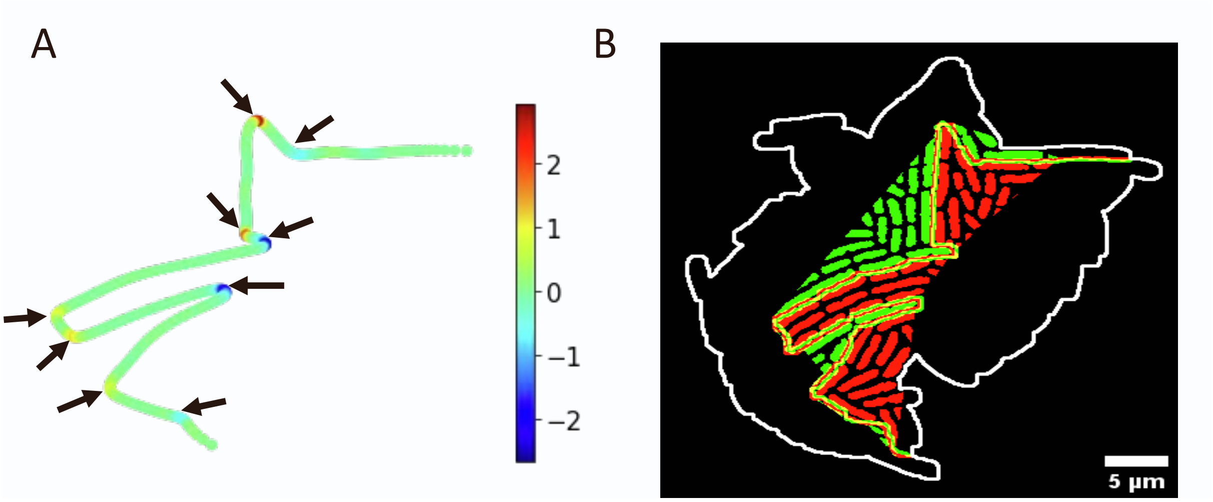

Supplementary Figure 11: Geometry of invasion and interfacial curve. A. Colormap of the signed curvature of the enclave interface curve computed using kappa plugin. Black arrows mark high curvature regions on the interface. **B.** Using high curvature points which marked the beginning of invasion front, we compute the region of invasion of one enclave into other.

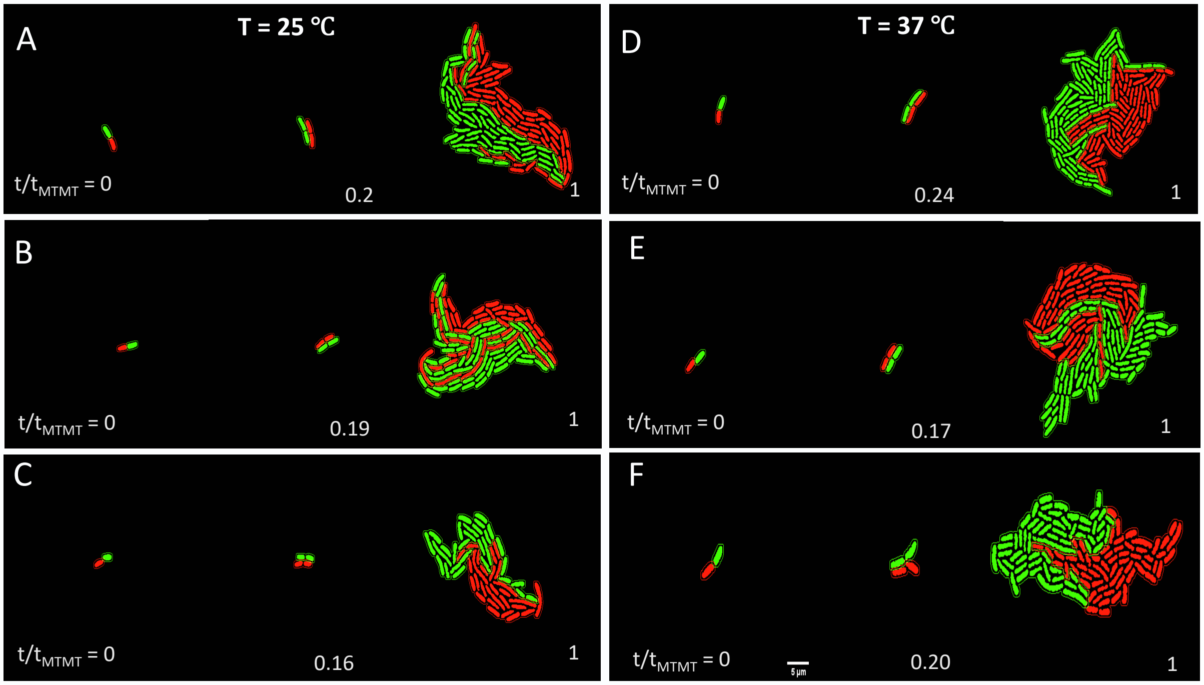

Supplementary Figure 12: Enclave formation is independent of arrangement of cells at the early stages of colony formation. Initial arrangement ($N_r = 2$, $N_g = 2$) of the two descendant cells is similar for the three replicas, shown for a representative colony growing at 25 °C (**A-C**) and at 37 °C (**D-F**). The final cell arrangement geometry for both the temperatures is clearly independent of the initial arrangement, in all cases.

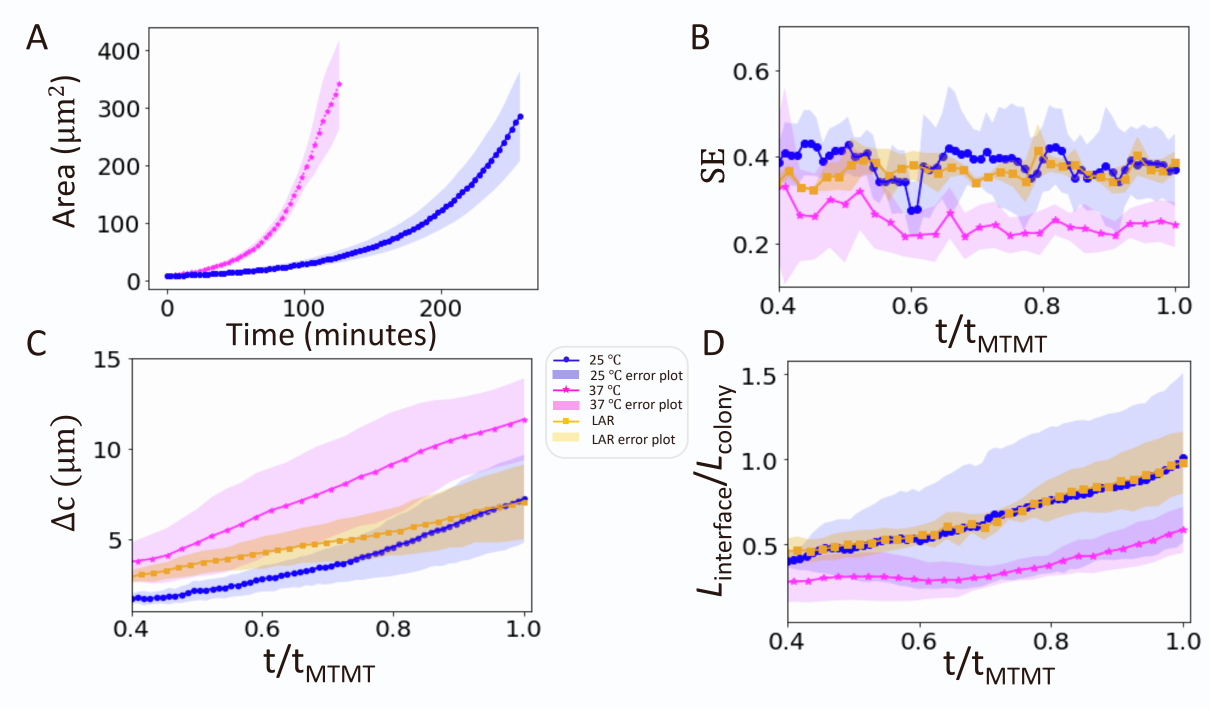

Supplementary Figure 13: Cell arrangement patterns are consistently more disordered for slower growing cells, display a similarity with cell arrangement patterns in colonies of low aspect ratio cells. A. The areal growth curve of the progeny enclaves is shown for *E.coli* cells growing at 37 °C (magenta) and 25 °C (blue). **B-D.** show comparison of time series of Shannon entropy (SE), relative distance between the centroids of the two enclaves (Δc) and normalised interfacial length ($L_{interface}/L_{colony}$) for *E.coli* colonies growing at 37 °C (magenta (stars)) and 25 °C (blue (circles)), and colonies formed by LAR cells growing at 25 °C (orange (squares)).

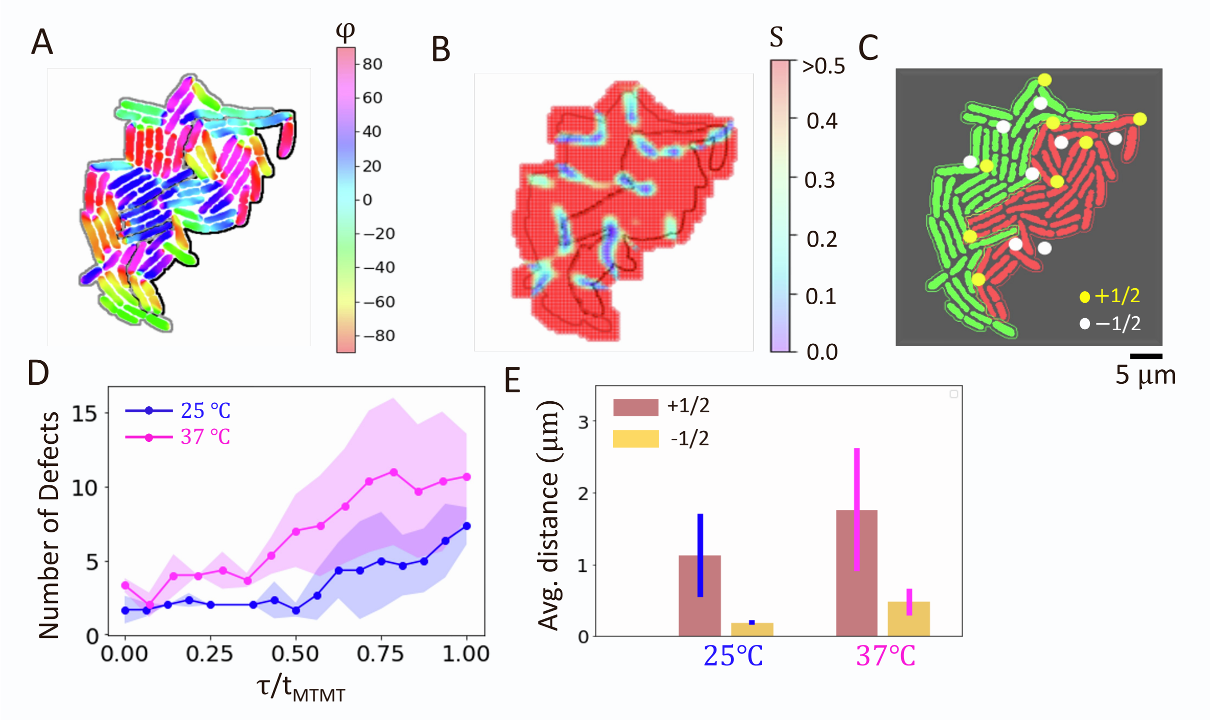

Supplementary Figure 14: Detection and tracking of topological defects. **A.** Colormap of orientation of cells in a representative colony with the enclave interfacial curve marked in black depicting that the majority of cells in the interface are aligned tangentially, **B.** Colormap of the nematic order parameter S, **C.** Topological defects of charge $+1/2$ (yellow dots) and $-1/2$ (white dots) marked out in the colony, with progeny enclaves colored red and green, **D.** Total number of defects for colonies growing at 25 °C (blue) and 37 °C (magenta) as a function of normalised time, **E.** Average distance travelled by $+1/2$ defects and $-1/2$ defects in colonies growing at 25 °C and 37 °C.

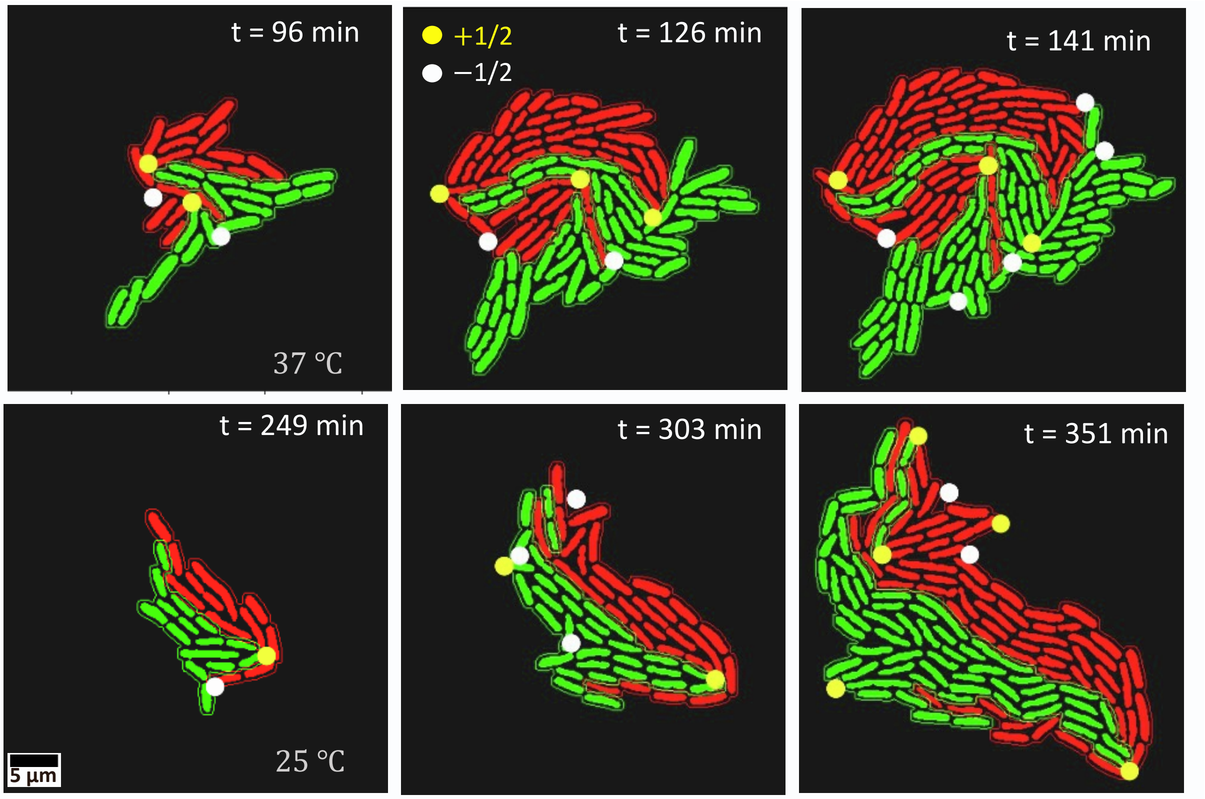

Supplementary Figure 15: Distribution of topological defects in bacterial colonies. Topological defects in representative colonies growing at 37 °C (up) and 25 °C (down) as they evolve with time. Note the proliferation of defects along the interface boundary.

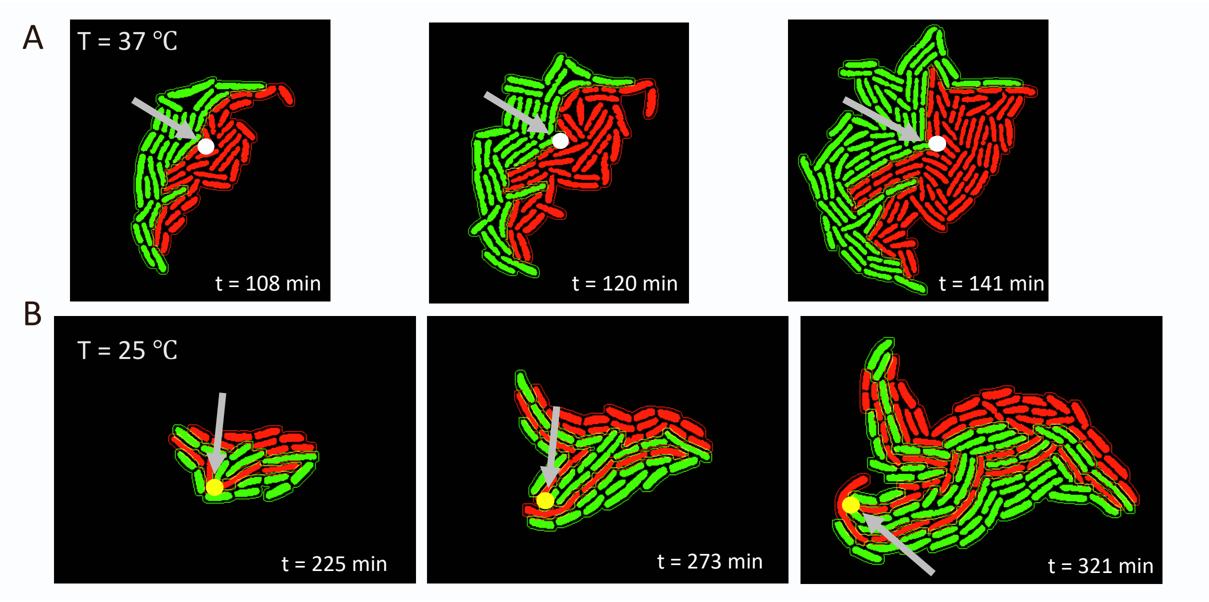

Supplementary Figure 16: Topological defects initiate enclave invasion and persist as invasion deepens. A. Initiation of a wide front enclave invasion nucleated by a $-1/2$ defect, which is marked by the arrow (left), with the defect persisting as the invasion front deepens in a colony growing at 37 °C (right). **B.** Initiation of a narrow front invasion nucleated a $+1/2$ defect (left) with the defect persisting as the invasion deepens in a colony growing at 25 °C (right).

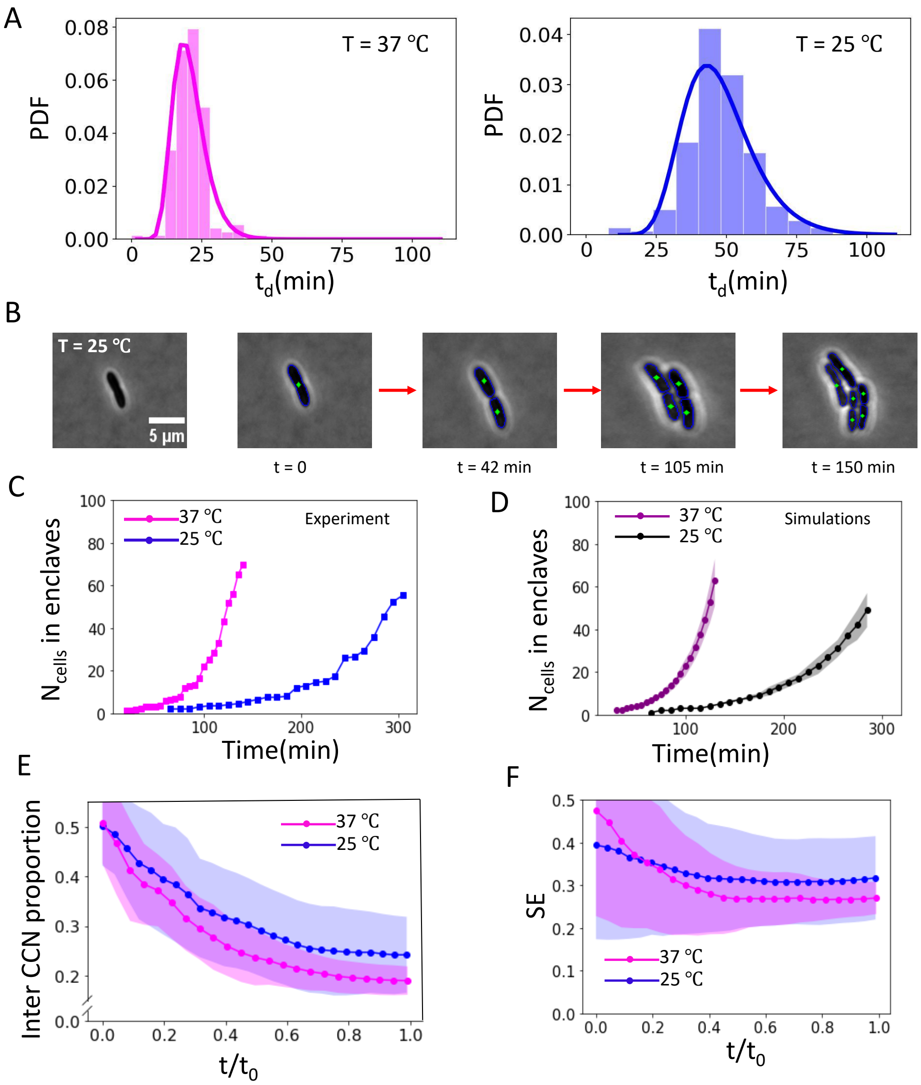

Supplementary Figure 17: Distribution of division times of cells in colonies, cell number evolution in colonies and analysis of simulated colonies. A. Normalized frequency for cell division time is plotted by tracking the division times of the cells in the colony as it evolves. The mean and standard deviation of the division times calculated from all replicas is $t_d = 21 \pm 6$ minutes at 37 °C and $t_d = 48 \pm 13$ minutes at 25 °C and the data was fitted with appropriate log normal distributions. **B.** Illustrative example of division time of the founder cell and subsequent daughter cells as they grow and divide at 25 °C. **C.** Growth of number of cells in colonies growing at 25 °C and 37 °C, observed in our experiments, **D.** Growth of number of cells in simulated colonies from our lattice model simulations. **E.** Proportion of Inter-enclave contacts for simulated colonies as function of normalised time, **F.** Shannon entropy of arrangement patters for simulated colonies as a function of time.

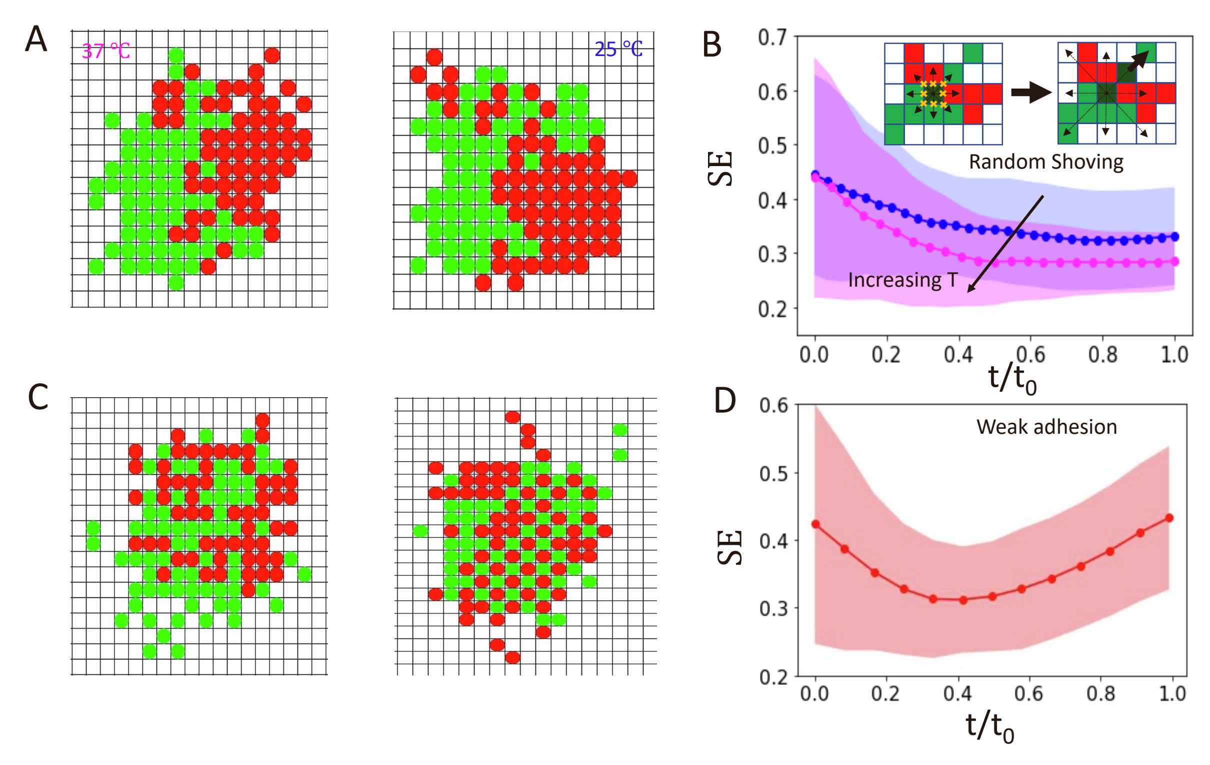

Supplementary Figure 18: Random cell shoving model and weakened daughter cell interaction model. **A.** Colonies simulated using cell shoving where direction is chosen randomly with equal probability, **B.** The evolution of Shannon entropy of arrangement with normalised time for faster growing (magenta) and slower growing (blue) colonies (INSET)- Schematic of random cell shoving of a dividing cell whose Moore neighbourhood is filled up, **C.** Snapshots of simulated colonies where one daughter cell was allowed to be placed away from neighbourhood of the mother cell (in particular two lattice steps away), thus not requiring two daughter cells to always be neighbours, in effect weakening the adhesion of daughter cells, **D.** Evolution of Shannon entropy of arrangements with normalised time for this case.

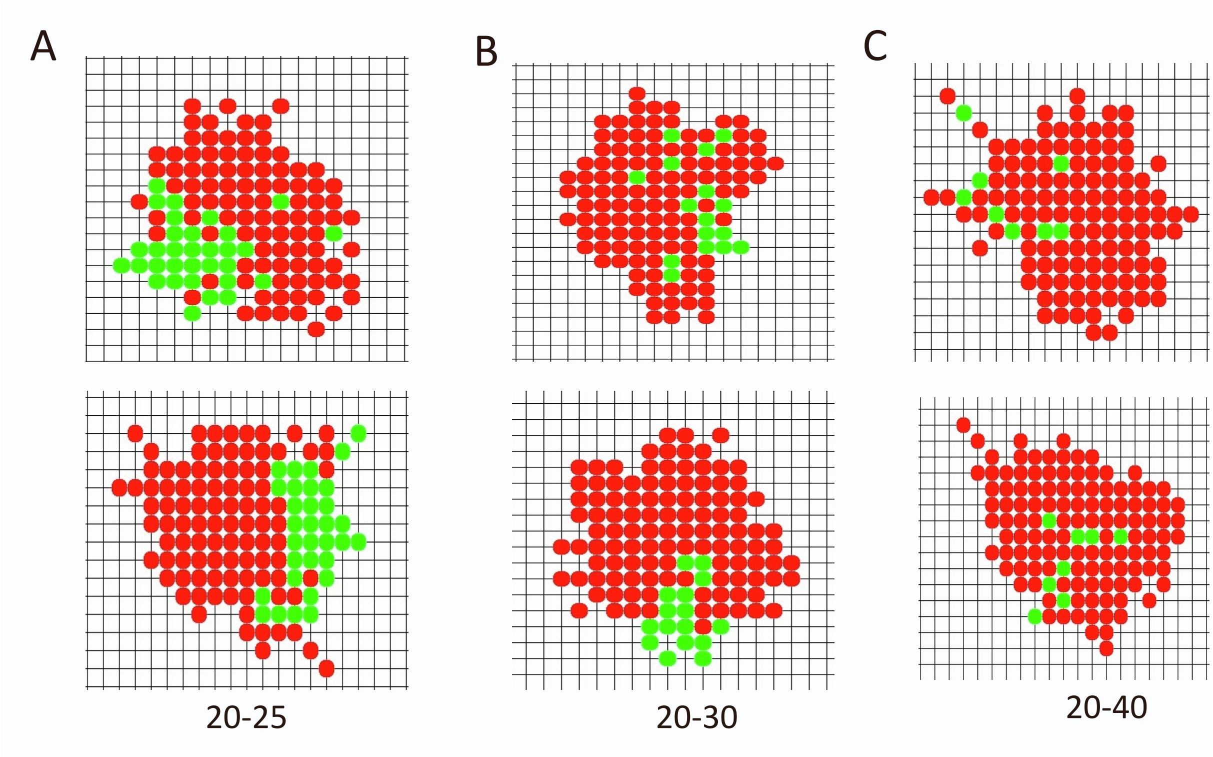

Supplementary Figure 19: Simulations of colonies comprising cells having different division times. Lattice model simulations of colonies starting from two initial cells where one cell and its descendants divide at 20 minutes and the other cell and its descendants divide at 25 mins (**A**), 30 mins (**B**) and 40 mins (**C**). While in (**A**) formation of progeny enclaves is clear, for (**B**) in some cases enclaves are formed while in other cases much larger degree of intermixing occurs. In (**C**), enclaves are almost always absent.
